# Distribution of *Dermacentor silvarum* and Associated Pathogens: Meta-Analysis of Global Published Data and a Field Survey in China

**DOI:** 10.3390/ijerph18094430

**Published:** 2021-04-22

**Authors:** Wen-Bin Guo, Wen-Qiang Shi, Qian Wang, Yu-Sheng Pan, Qiao-Cheng Chang, Bao-Gui Jiang, Jing-Xia Cheng, Xiao-Ming Cui, Yu-Hao Zhou, Jia-Te Wei, Yi Sun, Jia-Fu Jiang, Na Jia, Wu-Chun Cao

**Affiliations:** 1 State Key Laboratory of Pathogen and Biosecurity, Beijing Institute of Microbiology and Epidemiology, Beijing 100071, China; gwbin_2018@163.com (W.-B.G.); wqshi_mail@163.com (W.-Q.S.); w_qian1217@163.com (Q.W.); panys2046@163.com (Y.-S.P.); jiangbaogui@hotmail.com (B.-G.J.); cuixm7@163.com (X.-M.C.); zhouyuhao1994@126.com (Y.-H.Z.); weijiate7699@163.com (J.-T.W.); sunyi7310@sina.com (Y.S.); jiangjf2008@gmail.com (J.-F.J.); 2 Institute of EcoHealth, School of Public Health, Cheeloo College of Medicine, Shandong University, Jinan 250012, China; 3 College of Animal Science and Veterinary Medicine, Heilongjiang Bayi Agricultural University, Daqing 163319, China; changqiaocheng2001@163.com; 4 Department of Vector Control, Shanxi Provence Center for Disease Control and Prevention, Taiyuan 030012, China; chengjingxia007@163.com

**Keywords:** *Dermacentor silvarum*, ticks, tick-borne diseases, pathogens, geographical distribution

## Abstract

*Dermacentor silvarum* is an obligate blood sucking arthropod and transmits various pathogens to humans and domestic animals. Recently several new viruses were detected in *D. silvarum* as an emerging disease threat. In this study, we aimed to analyze its geographical distribution and associated pathogens. Data were collected from multiple sources, including a field survey, reference book, and literature review. We searched various electronic databases with the terms “*Dermacentor silvarum*” OR “*D. silvarum*” for studies published since 1963 and the positive rates for *Dermacentor silvarum*-associated pathogens were estimated by meta-analysis. *D. silvarum* was found only in four countries in Eurasia, ranging from 22° N to 57° N latitude. At least 20 human pathogens were associated with *D. silvarum*, including five species of spotted fever group rickettsiae, three species in the family of Anaplasmataceae, three genospecies in the complex *Borrelia burgdorferi* sensu lato, *Francisella tularensis, Babesia venatorum, Coxiella buenetii, Borrelia miyamotoi,* and five species of virus. Among them, *Rickettsia raoultii* was widely detected in *D. silvarum*, showing the highest pooled positive rate (25.15%; 95% CI 13.31–39.27). Our work presents the most comprehensive data and analysis (to our knowledge) for the geographical distribution of *D. silvarum* and associated pathogens, revealing an emerging threat to public health and stocking farming. Continued surveillance and further investigations should be enhanced.

## 1. Introduction

Ticks are obligate blood-sucking ectoparasites distributed widely across the world. They are vectors of many human and animal infectious pathogens, such as tick-borne encephalitis, Crimean-Congo hemorrhagic fever, Lyme disease, Q fever, babesiosis, and severe fever with thrombocytopenia syndrome [1,2,3]. Currently, tick-borne diseases have become an increasing public health threat with global climate changing, accelerated urbanization, and altered distribution of tick and their hosts [4,5,6].

*Dermacentor silvarum*, a species of hard ticks, is widely distributed in North China, Russia, and Mongolia [7,8,9]. They have a hard scutum, the cornua of basis capituli is longer and pointy, and the length is nearly equal to the base width. The dorsal spur of trochanter I is long and pointed, and the apex of the third segment of the pedipalp is flat [10] (Figure S1). They mainly reside in shrubbery, and sometimes can also be found in forests and farmland [11]. The immature ticks mainly infest a variety of small mammals, and adults feed on medium-sized to large mammals, including humans [8]. It has been reported that *D. silvarum* can carry a large variety of tick-borne pathogens including tick-borne encephalitis virus, *Rickettsia slovaca, R. raoultii, Anaplasma phagocytophilum, Babesia caballi,* and *Theileria equi*, as well as the causative agent of human monocytic ehrlichiosis, *Ehrlichia chaffeensis* [12,13,14,15]. Moreover, with the development of high throughput sequencing, lots of novel tick-borne viruses have been discovered in *D. silvarum* in recent years, such as Tacheng tick virus 1 and Jingmen tick virus [16,17].

It is of great importance to know the distribution of *D. silvarum* and its associated pathogens. Previous studies have depicted the distribution of *D. silvarum* in China and in Eurasia [18,19]. Here, we comprehensively collected the geographical distribution data of *D. silvarum* and its associated pathogens, and conducted a meta-analysis to assess its potential threat for public health.

## 2. Materials and Methods

### 2.1. Data Collection and Field Survey

Data were collected from multiple sources, including a field survey, reference book, and literature. The field survey was carried out in all provinces, autonomous regions, and municipalities of mainland China from April to November in 2016–2019. Questing ticks were collected by dragging a standard 1-m^2^ flannel flag over vegetation, and infesting ticks were collected from body surfaces of animals (mainly domestic animals). All ticks were transported alive or preserved in 75% alcohol to our laboratory for taxonomic identification. Tick species were identified based on morphological features [10] by an entomologist, and all identified *D. silvarum* ticks were included in this study. Locations of survey sites are listed in Table S1. All experimental procedures and protocols were approved by the Ethics Committee of Academy of Military Medical Sciences (Permit Number: 2016YFC1201902).

Using the terms “*Dermacentor silvarum*” OR “*D. silvarum*” (and the Chinese name for *Dermacentor silvarum*), we searched multiple electronic databases, including PubMed, China National Knowledge Infrastructure (CNKI), and the WanFang database. The study period was 1 January 1963 to 15 December 2020. A publication (a journal article, conference proceeding, or degree thesis) was retrieved if the terms appeared in any parts of its content. Published full articles were included if they were in English or Chinese and had reported the collection locations of *D. silvarum* on a county or prefecture level (some Russian papers might be missed). Articles which report only laboratory findings, describe identification of tick-borne diseases, or do not include any geographical information were excluded. The key information extracted from the literature included: (i) location associated with tick occurrence (and its geographical scale), (ii) time of tick collection, (iii) species of pathogens identified (if any), and (iv) number of tests and number of positives. After the data were collected, a second person checked the dataset thoroughly to avoid errors and duplications. We also included the distribution data of *D. silvarum* from the reference book, *Fauna Sinica-Arachnida Ixodida* by YS [20]. All the data from literature search and our field survey are provided in Table S1.

### 2.2. Distribution of D. Silvarum and Associated Pathogens

To map geographical distribution of *D. silvarum* and associated pathogens, we obtained the raster-type data with a resolution of one kilometer on vegetation types from the China National Forestry Bureau and a global map of altitude topography. Thematic maps were produced with ArcGIS 10.7 software (ESRI Inc., Redlands, CA, USA). The particular geographical locations of *D. silvarum* were used for mapping, and the administrative region centroids were used when exact locations were not available.

We did a meta-analysis to estimate the combined positive rate and 95% CI of each *D. silvarum*-associated pathogen. Studies without exact numbers of ticks were excluded. If there was only one study included for a certain species of pathogen, the positive rate was calculated by dividing the number of positive ticks by total number without a 95% CI. If the number of studies was two or more, the combined positive rate and 95% CI were estimated. Given the data variance in multiple studies, we chose the random effects model for our meta-analysis [21]. The meta-analysis was done with R (version 3.6.0, meta package).

## 3. Results

### 3.1. Dataset

**Figure 1:**
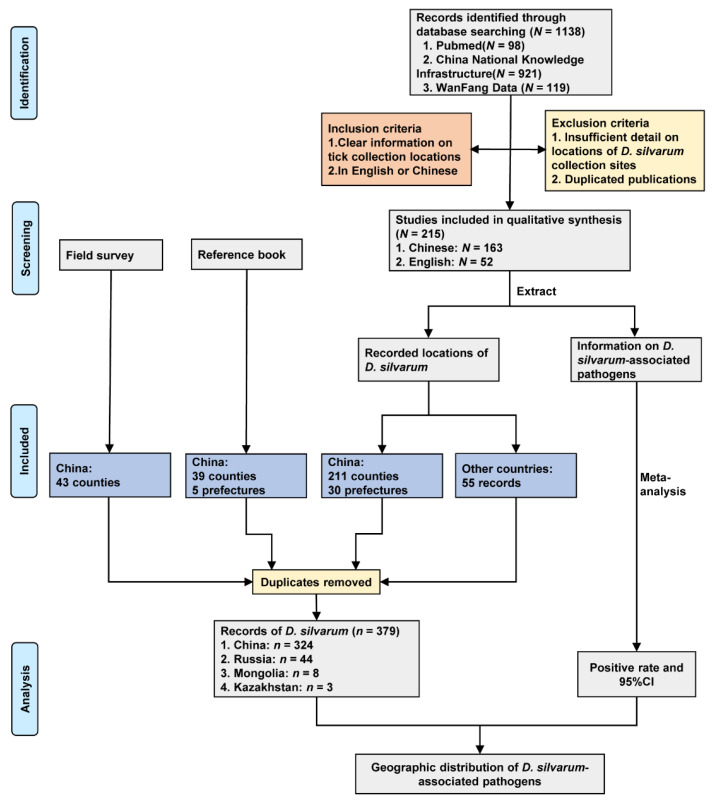
Study design and data sources. Data were collected from a field survey, reference book and literature review. *N* indicates the number of studies, and *n* represents the number of records of *D. silvarum*.

We obtained 43 records from field survey, 40 records from the reference book, and 296 records from publications. After removing duplicate locations, there were 379 records of *D. silvarum* in four countries (Figure 1), including China (324 records), Russia (44 records), Mongolia (8 records), and Kazakhstan (3 records).

### 3.2. Distribution of Dermacentor Silvarum

**Figure 2:**
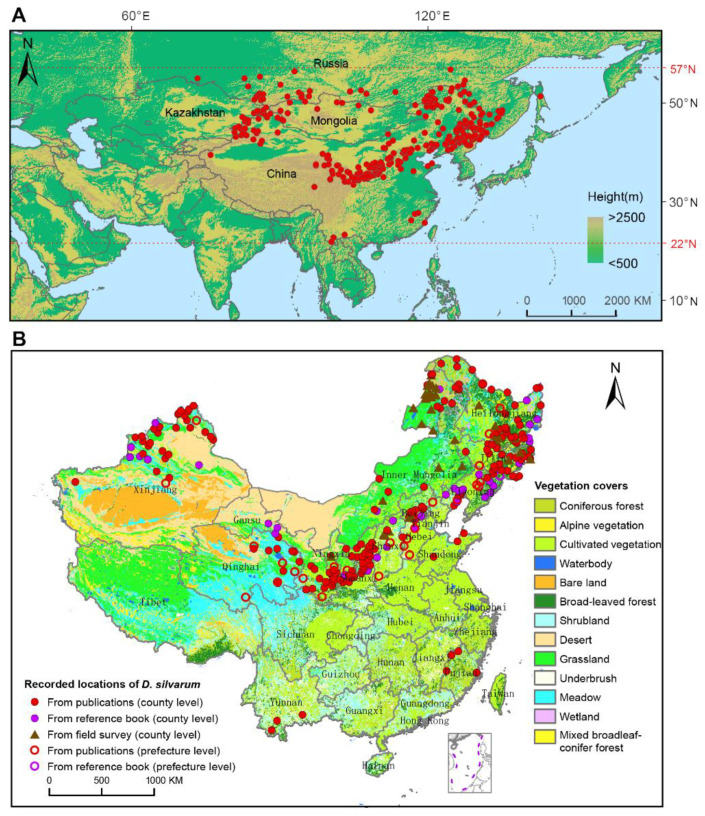
Geographical distribution of *Dermacentor silvarum*. (**A**) Recorded locations of *D. silvarum* worldwide on an elevation map marked with red dots. It was found to be distributed only in Eurasia, ranging from 22° N to 57° N latitude. (**B**) Recorded locations of *D. silvarum* and vegetation coverage in China. Dots represent the county-level regions, and circles represent the prefecture-level regions.

*D. silvarum* was found only in the northern hemisphere, ranging from 22° N to 57° N latitude (Figure 2A). The easternmost location was the Pacific coast in the southern Sakhalin [9]. The southernmost location was as far as 22° N latitude, which was recently confirmed by Liu et al. [22]. The site of 73.36° E/54.99° N in Omsk, Russia, was documented as the westernmost location [9]. The northernmost distribution could be reasonably defined as the southern districts of the Yakutia Republic, Russia, where *D. silvarum* was observed up to 56.8° N. *D. silvarum* was mainly distributed in high altitudinal areas around the mountains. In Russia, *D. silvarum* was found in the highland of southern Siberia, including the Altai, the mountains around Lake Baikal and the Stanovoy Range. In the Far East, the locations of *D. silvarum* were connected to the locations in northeastern China. In Mongolia, *D. silvarum* was recorded in the north region, surrounding the Khentii mountains, and in the southwest area, close to the Altai Mountains. In Kazakhstan, *D. silvarum* was documented in three locations, which also belong to the Altai Mountains (Figure S2).

Figure 2B shows the spatial distribution of *D. silvarum* and vegetation coverage in China. *D. silvarum* was detected in 11 provinces, three autonomous regions, and one municipality, presenting in 290 counties and 34 prefectures. *D. silvarum* was mostly reported in the north of China, but it can be found in southeast of China (Fujian province) and southwest of China (Yunnan province). The distribution of *D. silvarum* was usually close to mountain ranges, including Greater Khingan Mountains, Taihang Mountains, Qinling Mountains, Qilian Mountains, and Tianshan Mountains. In addition, *D. silvarum* prefers broad-leaved forest, coniferous forest, cultivated vegetation, and shrubland vegetations. The distribution of *D. silvarum* in different periods of years in China is shown in Figure S3. It reveals a permanent distribution in the north of China, especially in northeast China. Interestingly, before 2000, *D. silvarum* was sporadically documented in Guangze county, Fuqing County, Ninghua County, and Wuyi Mountain in Fujian Province, but disappeared in the record after 2000. In contrast, *D. silvarum* had never been reported in Yunnan province until 2019 [22]. However, we only collected *D. silvarum* in 6 provinces or autonomous regions, despite fieldwork in most parts of mainland China (Figure S4; Table S1). A total of 4268 *D. silvarum* were collected, most of which were collected in April and May, each year. About 40% of the *D. silvarum* were free adult ticks, and the hosts were mainly domestic animals, cattle, goat, and sheep.

### 3.3. Dermacentor Silvarum-Associated Pathogens

**Figure 3:**
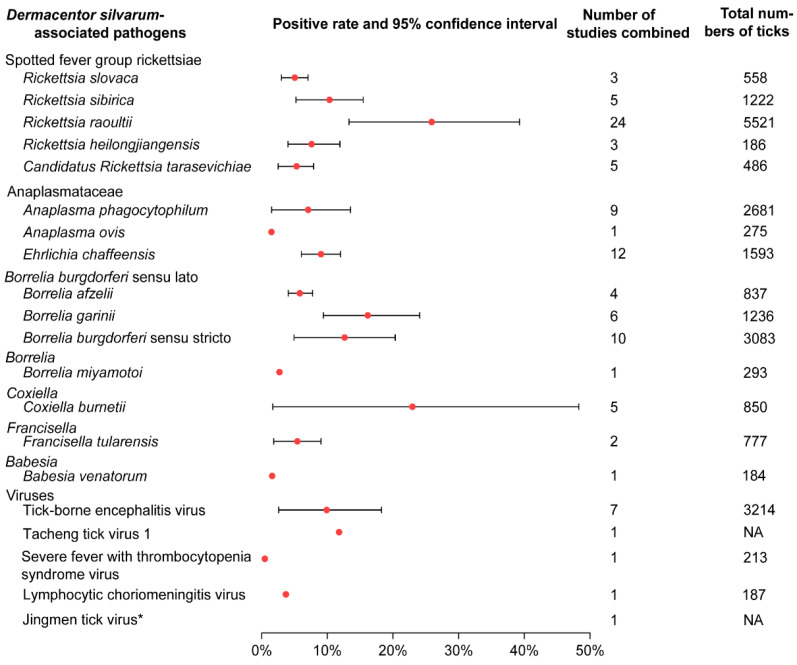
Prevalence of pathogens associated with *Dermacentor silvarum*. If there was only one study included for a certain pathogen, CI was not calculated. CI: Confidence Interval. * indicates the virus detected by deep sequencing, and the original article did not give the specific number of tests and positive numbers. NA: no date.

There were 20 human pathogens detected in *D. silvarum* (Figure 3; Table S2). Among them, five species were spotted fever group rickettsiae (SFGR). *Rickettsia raoultii* showed the highest pooled positive rate (25.15%; 95% CI 13.31–39.27), while *Candidatus Rickettsia tarasevichiae* showed the lowest (4.12%; 95% CI 1.08–7.15; Table S3). There were three species in the family of Anaplasmataceae, and the positive rate of *E. chaffeensis* was 9.03% (95% CI 6.04–12.01), relatively higher than *A. phagocytophilum* (6.14%; 95% CI 1.53–13.54) and *A. ovis* (1.5%). Three genospecies were detected in the complex *Borrelia burgdorferi* sensu lato (s.l.), among which the pooled positive rates of *B. garinii* and *B. burgdorferi* sensu stricto (s.s.) were 15.03% (95% CI 9.39–24.08) and 12.64% (95% CI 4.93–20.35), respectively. Five species of the viruses were detected in *D. silvarum*, while Tacheng tick virus 1 had the highest positive rate of 11.8%. Other pathogens *D. silvarum* harboured were *Francisella tularensis, Babesia venatorum, Coxiella buenetii*, and *Borrelia miyamotoi*, and the positive rates range from 1.6% to 19%. Additionally, animal pathogens, such as *Bartonella, Hepatozoon, Theileria*, and agents with unknown pathogenicity were also detected (Table S3; Figure S5).

**Figure 4:**
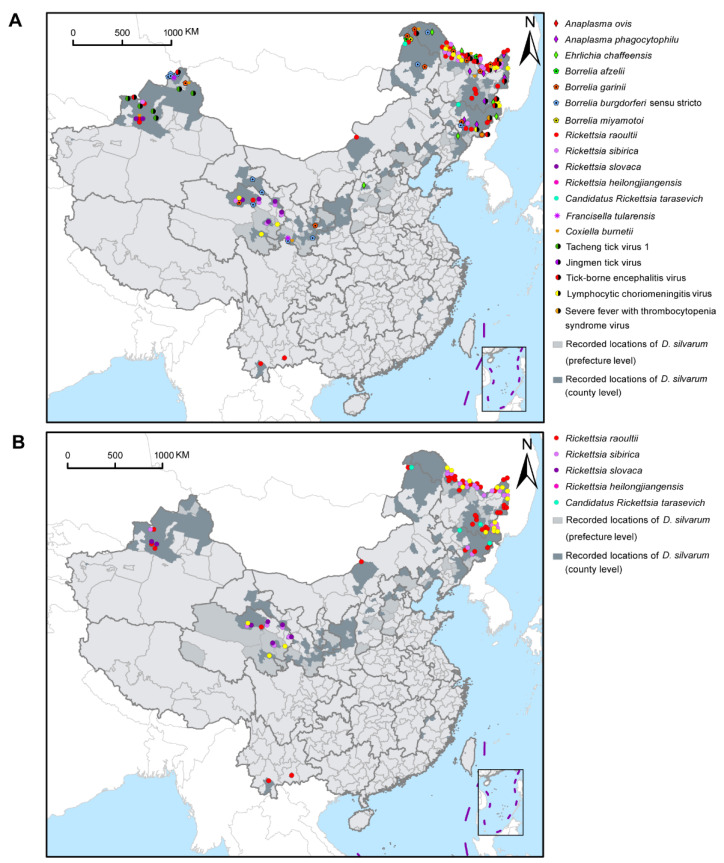
Geographical distribution of pathogens with *Dermacentor silvarum* in China. (**A**) Distribution of 20 human pathogens presented in *D. silvarum* in China. (**B**) Geographical distribution of five SFGR species detected in *D. silvarum* in China. *Rickettsia raoultii* was the predominant SFGR found in *D. silvarum* and widely distributed. SFGR: spotted fever group rickettsiae.

Figure 4A showed the geographical distribution of *D. silvarum*-associated human pathogens in China. Although *D. silvarum* was widely distributed in northern China, various pathogens were mainly reported in the northeast regions of China, including four species of SFGR, three species in the family Anaplasmataceae, three genospecies in the complex *B. burgdorferi* s.l., four species of the viruses, and *Coxiella burnetii*. Most species of SFGR and *Borrelia* were widely reported, especially *R. raoultii*, in northeast, central-north, northwest, and southwest areas of China (Figure 4B). Some other pathogens, however, were only detected in certain regions, for example, *R. slovaca* only reported in Xinjiang Uygur Autonomous Region and Qinghai province, Tacheng tick virus 1 in Xinjiang Uygur Autonomous Region, and Jingmen tick virus in Heilongjiang province, so far (Figure S6). In Russia, at least three human pathogens, including *R. raoultii*, *Candidatus Rickettsia tarasevichiae*, and tick-borne encephalitis virus, were sporadically distributed in the southern border region. Only *Ba. caballi* was reported in *D. silvarum* in Mongolia (Figure S7).

## 4. Discussion

This study confirmed previous distribution data, updating information with more recent reports, as well as a field survey carried out in China during 2016–2019. Our results showed that *D. silvarum* were present in four countries of the world, and the locations were usually surrounded with mountains within the belt of 22–57° N latitude. At least 20 human pathogens were carried by *D. silvarum*, including various species of *Rickettsia, Anaplasma, Borrelia*, and viruses.

Although the presence of a pathogen in a vector does not prove its transmission capability, the vector competence of *D. silvarum* for *R. raoultii* and Tick-borne encephalitis virus (TBEV) have been proved [23,24], as well as *R. sibirica* and *Ba. caballi* [25,26]. *R. raoultii, R. sibirica,* and TBEV as the causative agents of tick-borne lymphadenopathy, Siberian tick typhus, and tick-borne encephalitis, respectively, can cause the patient fever, headache, and other symptoms [27]. *Ba. caballi* as the causative agent of equine piroplasmosis, can cause haemolytic anaemia and abortions [28]. These pathogens can be transmitted by *D. silvarum* and cause severe harm to human health, as well as economic loss to livestock production.

It is well known that the spatial distributions of ticks are influenced by many factors, including host availability, vegetation, ambient temperatures, and other abiotic and biotic environmental conditions [29].The life cycle of *D. silvarum* takes approximately one year. The egg hatching starts when the mean ambient temperature is higher than 16 °C, and is active from late February to early September under field conditions. Then, adults enter a behavioral diapause, which lasts until the following spring [30]. In our study, *D. silvarum* was mainly clustered in northern China and southern Russia, where most climate types are boreal (continental) climates, especially boreal climates with precipitation all year round and boreal climates with dry winters [19]. The oviposition of females and egg hatching was synchronized, irrespective of the month in which the females engorged to ensure that the emergence of eggs and larvae coincide with optimal conditions for development. In fact, *D. silvarum* is particularly well adapted to cold winter temperatures; the lowest temperature points (at which body fluids spontaneously freeze) of larvae and adults averaged −20.0 °C and −23.9 °C, respectively [31]. Moreover, unfed adults can enter a behavioral diapause, remaining quiescent under the leaf litter without actively questing for host, which can last about 9–10 months to over winter [30]. All of these may help it survive the cold boreal climates.

Figure 2B shows that the *D. silvarum* mainly reside in broad-leaved forest, coniferous forest, cultivated vegetation, and shrubland, most of which are in the high altitudinal regions. *D. silvarum* is a three-host tick species with a broad host range, involving domestic and wild animals. In our field survey and literature search, we found that the adults mainly feed on domestic goats, cattle, horses, and wild-boar, while the immature ticks prefer to infest a variety of small mammals, such as rodents, hares, and hedgehogs [11]. Obviously, there could be a correlation between these hosts and the habitats. In the high altitudes surrounding the mountains, it can provide a suitable environment and easy access to a blood meal.

Although, several species of ticks have been expanding their geographical ranges in recent decades, largely due to climate change [29,32], the distribution of *D. silvarum* does not seem to be significantly changed in China (Figure 2B; Figure S3). It is noteworthy that *D. silvarum* occurred sporadically in Fujian and Yunnan provinces, southeast and southwest China, respectively. In Fujian province, *D. silvarum* was documented in four locations next to the Wuyi mountains [33,34,35]. Moderate temperature, rich vegetation species, and suitable hosts could explain its existence. However, all records of *D. silvarum* in Fujian province were around 20 years ago, and further investigation should be performed. The appearance of *D. silvarum* in Yunnan province was possibly linked to birds’ migration, highlighting the possibility of *D. silvarum* transported on host and subsequently establishing in new areas. Liu et al. [22] collected 56 free adult *D. silvarum* in three locations of Yunnan province in 2019, where covering with forest and meadow steppe favor a range of wild and domestic animals and provide suitable habitats [36]. The report of *D. silvarum* in new areas may pose a significant public health problem. Further work on its genome sequence may be able to shed some light on where it came from and how it transported here. As our field survey in Fujian and Yunnan were conducted in September, the peak period might be missed [8].

We identified 38 agents carried by *D. silvarum* globally, among which at least 20 are pathogenic to humans. Most species of *Rickettsia* carried by *D. silvarum* are human pathogens. *R. raoultii* was the predominant *Rickettsia* found in *D. silvarum* of China and Russia, with wide distribution and high infection rate (25.15%; 95% CI 13.31–39.27). *R. raoultii*, as the causative agent of tick-borne lymphadenopathy or *Dermacentor*-borne necrosis erythema lymphadenopathy [37,38], could be maintained in *Dermacentor* ticks for 4–7 generations with a high level of transovarial and transstadial transmission [23]. So, *D. silvarum* may play a major role in transmission of *R. raoultii* to human, and a patient bitten by *D. silvarum* and with an inoculation eschar on the scalp or cervical lymphadenopathies should be suspected with *R. raoultii* infection. TBEV, with the positive rate of 8.85% (95% CI 2.62–18.26) in *D. silvarum* in China and Russia, can attack the human central nervous system, leading to tick-borne encephalitis [39]. Although *Ixodes persulcatus* and *I. ricinus* are the main TBEV vectors, *D. silvarum* has also been proven to be the vector [24]. Therefore, in northeast China and the far east of Russia more attention should be paid to tick-borne encephalitis surveillance and prevention.

Lots of viruses were detected by next-generation sequencing (NGS) in *D. silvarum* in recent years, such as Tacheng tick virus 1, Jingmen tick virus, Blacklegged tick phlebovirus, and Deer tick Mononegavirales-like virus [16,17,40]. Tacheng tick virus 1 as a causative pathogen, has been isolated from a patient with fever and identified in several species of ticks, near the residence. JMTV as a segmented RNA virus have been identified in arthropods and mammals [41,42,43], and isolated from the tick, *Amblyomma javanense* [16], showing a remarkable diversity and a global geographical distribution. All these data reveal their potential pathogenicity to humans. As a new detection method, NGS is more efficient than traditional methods, and is helpful to promote the discovery of new pathogens in *D. silvarum* and other tick species. Further laboratory studies are required to test the potential pathogenicity and vector capacities of some other unexplored pathogens detected in *D. silvarum.*

The study has following limitations: first, articles published in languages other than Chinese and English were not included, which may lead to some data omissions about *D. silvarum* in some regions, especially in Mongolia and Russia. Second, it must also be considered that the absence of reports in some areas may be caused by lack of collection effort rather than true absence. Third, *D. silvarum* and associated pathogens have been reported to be identified at the China-North Korea border. According to the prevailing climate, it can be assumed that *D. silvarum* is also endemic in North Korea, although there is no evidence for this in the literature so far. Finally, the accuracy of risk assessment based on published data might be limited by the varying sensitivities of detection methods in each study.

## 5. Conclusions

In summary, we showed that *D. silvarum* was found only in four countries in Eurasia, ranging from 22° N to 57° N latitude, and at least 20 human pathogens were associated with *D. silvarum*. Our work presents the most comprehensive data and analysis (to our knowledge) for the geographical distribution of *D. silvarum* and its associated pathogens, some of which can cause human and animal diseases, and some are new pathogens, revealing an emerging threat to public health and stocking farming. It should be noted that some Russian papers might be missed in this study using our approach. Further surveillance and investigation should be strengthened in areas where *D. silvarum* and associated pathogens are presented.

## Data Availability

The data presented in this study are available on request from the corresponding author.

## Supplementary Materials

The following are available online at https://www.mdpi.com/article/10.3390/ijerph18094430/s1, Figure S1: *Dermacentor silvarum*, Figure S2: Geographical distribution of *Dermacentor silvarum* in Russia, Mongolia, and Kazakhstan, Figure S3: Recorded locations of *Dermacentor silvarum* in different periods of years in China, Figure S4: Map of field survey sites in China from 2016 to 2019, Figure S5: Meta-analysis of positive rate of each *Dermacentor silvarum*-associated agent, Figure S6: Geographical distribution of each *Dermacentor silvarum*-associated agent in China, Figure S7: Geographical distribution of *Dermacentor silvarum*-associated agents in Russia and Mongolia, Table S1: The dataset, Table S2: References of each agent for meta-analysis, Table S3: Prevalence and pathogenicity of *Dermacentor silvarum*-associated agents.
