# Supplementary material for: Distribution of Dermacentor silvarum and Associated Pathogens: Meta-Analysis of Global Published Data and a Field Survey in China
Source: Int J Environ Res Public Health. 2021 Apr 22;18(9):4430. doi: 10.3390/ijerph18094430 (PMC8122522; doi:10.3390/ijerph18094430)
Supplement: Supplementary file 1 [file ijerph-18-04430-s001.zip › Supplementary -pdf/FigureS5.pdf]

Figure S5. Meta-analysis of positive rate of each *Dermacentor silvarum*-associated agent.

Anaplasma phagocytophilum

Study	Events	Total	Proportion	95%−CI	Weight (fixed)	Weight (random)
Li et al. 2012	144	594	0.24	[0.21; 0.28]	22.2%	11.5%
Cheng et al. 2019	6	297	0.02	[0.01; 0.04]	11.1%	11.3%
Cheng et al. 2017	6	297	0.02	[0.01; 0.04]	11.1%	11.3%
Cheng et al. 2015	4	56	0.07	[0.02; 0.17]	2.1%	10.3%
Wei et al. 2016	5	204	0.02	[0.01; 0.06]	7.6%	11.2%
Cao et al. 2006	2	286	0.01	[0.00; 0.03]	10.7%	11.3%
Zhang et al. 2012	143	694	0.21	[0.18; 0.24]	25.9%	11.5%
Dong et al. 2013	5	61	0.08	[0.03; 0.18]	2.3%	10.4%
Jiang et al. 2011	6	192	0.03	[0.01; 0.07]	7.2%	11.2%
**Fixed effect model**		**2681**	**0.10**	**[0.08; 0.11]**	**100.0%**	**--**
**Random effects model**			**0.06**	**[0.02; 0.14]**	**--**	**100.0%**

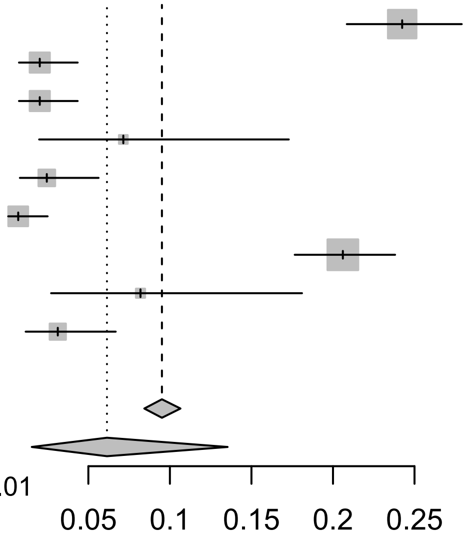

Heterogeneity: $I^2 = 98\%$, $\tau^2 = 0.0359$, $p < 0.01$

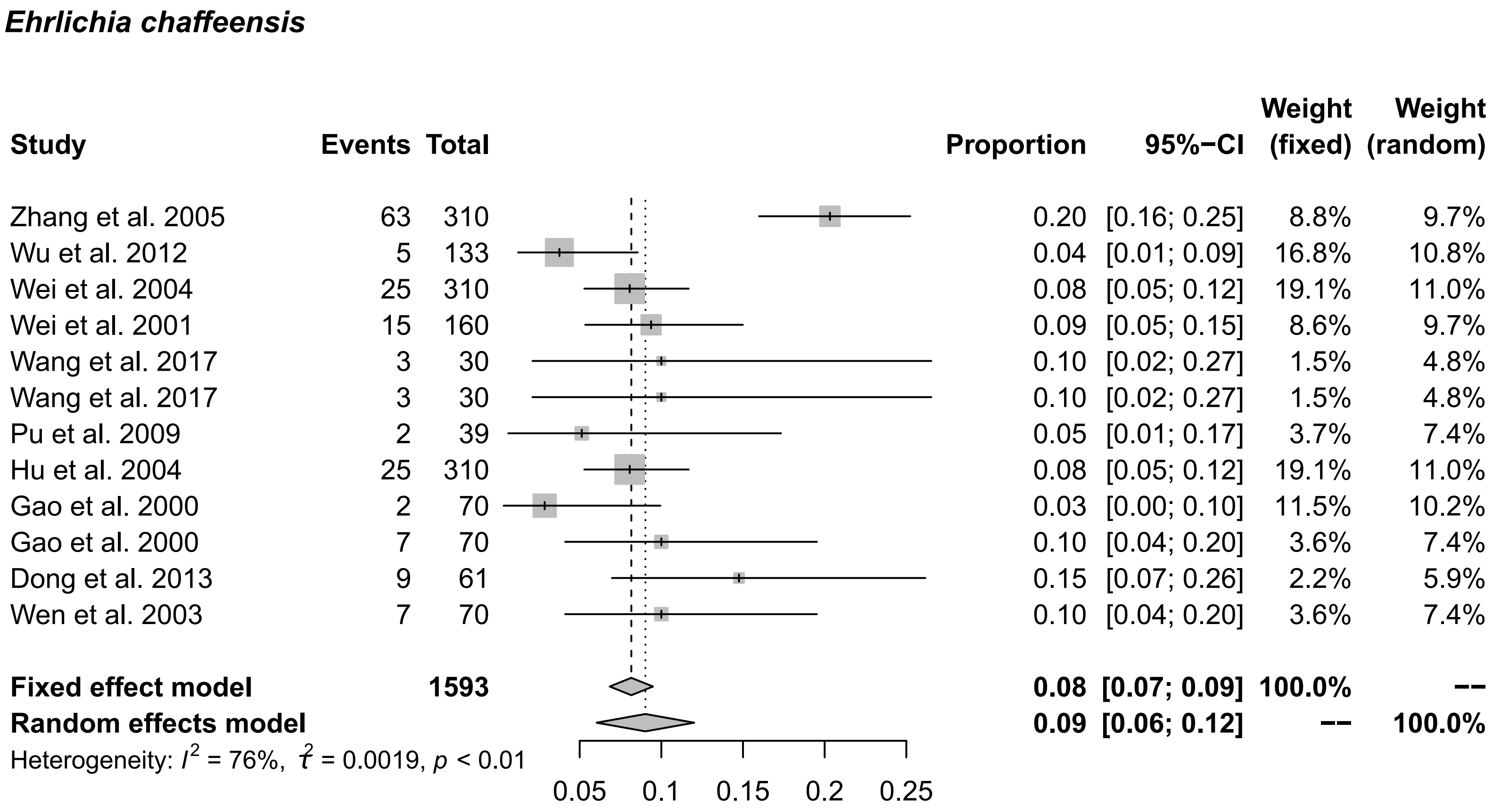
Ehrlichia chaffeensis
Study
Events
Total
Proportion
95%−CI
Weight (fixed)
Weight (random)
Zhang et al. 2005 63 310 0.20 [0.16; 0.25] 8.8% 9.7%
Wu et al. 2012 5 133 0.04 [0.01; 0.09] 16.8% 10.8%
Wei et al. 2004 25 310 0.08 [0.05; 0.12] 19.1% 11.0%
Wei et al. 2001 15 160 0.09 [0.05; 0.15] 8.6% 9.7%
Wang et al. 2017 3 30 0.10 [0.02; 0.27] 1.5% 4.8%
Wang et al. 2017 3 30 0.10 [0.02; 0.27] 1.5% 4.8%
Pu et al. 2009 2 39 0.05 [0.01; 0.17] 3.7% 7.4%
Hu et al. 2004 25 310 0.08 [0.05; 0.12] 19.1% 11.0%
Gao et al. 2000 2 70 0.03 [0.00; 0.10] 11.5% 10.2%
Gao et al. 2000 7 70 0.10 [0.04; 0.20] 3.6% 7.4%
Dong et al. 2013 9 61 0.15 [0.07; 0.26] 2.2% 5.9%
Wen et al. 2003 7 70 0.10 [0.04; 0.20] 3.6% 7.4%
Fixed effect model 1593 0.08 [0.07; 0.09] 100.0% --
Random effects model 0.09 [0.06; 0.12] -- 100.0%
Heterogeneity: I^2 = 76%, τ^2 = 0.0019, p < 0.01
0.05
0.1
0.15
0.2
0.25

Babesia caballi

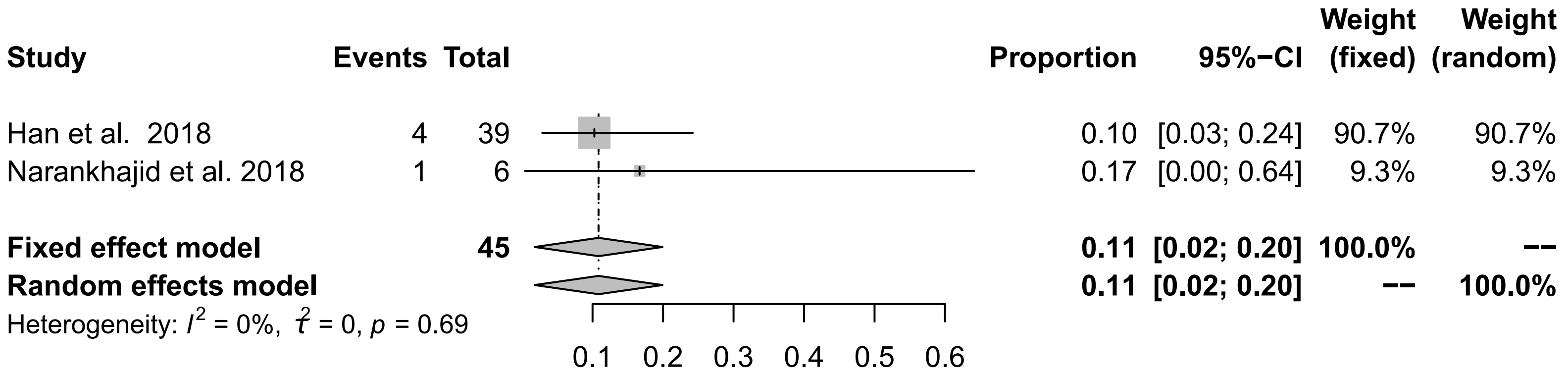

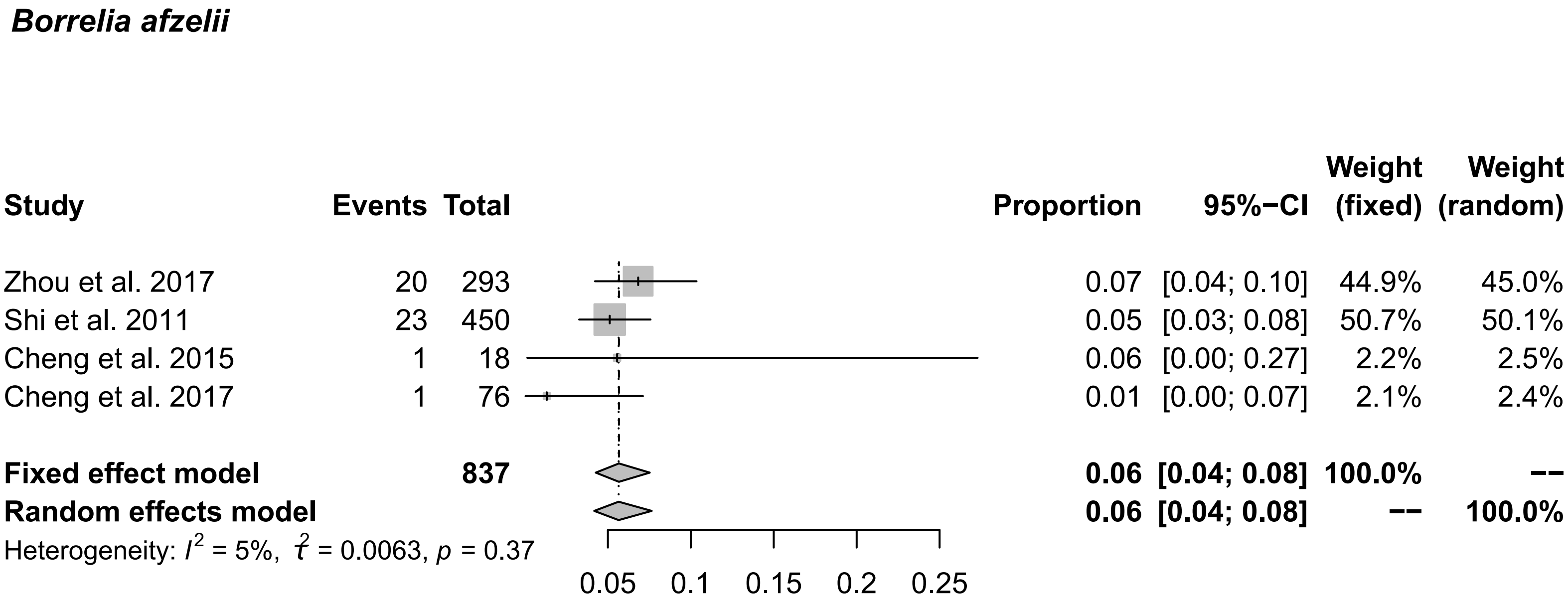

Borrelia afzelii
Study
Events
Total
Proportion
95%−CI
Weight (fixed)
Weight (random)
Zhou et al. 2017 20 293 0.07 [0.04; 0.10] 44.9% 45.0%
Shi et al. 2011 23 450 0.05 [0.03; 0.08] 50.7% 50.1%
Cheng et al. 2015 1 18 0.06 [0.00; 0.27] 2.2% 2.5%
Cheng et al. 2017 1 76 0.01 [0.00; 0.07] 2.1% 2.4%
Fixed effect model 837 0.06 [0.04; 0.08] 100.0% --
Random effects model 0.06 [0.04; 0.08] -- 100.0%
Heterogeneity: I^2 = 5%, τ^2 = 0.0063, p = 0.37
0.05
0.1
0.15
0.2
0.25

***Borrelia burgdorferi* sensu stricto**

**Study**	**Events**	**Total**	**Proportion**	**95%−CI**	**Weight (fixed)**	**Weight (random)**
Zhang et al. 2011	82	394	0.21	[0.17; 0.25]	0.1%	10.2%
Luo et al. 2011	15	126	0.12	[0.07; 0.19]	0.1%	9.9%
Liu et al. 2000	13	87	0.15	[0.08; 0.24]	0.0%	9.5%
Liu et al. 1992	1	36	0.03	[0.00; 0.15]	0.1%	10.0%
Huang et al. 2006	101	327	0.31	[0.26; 0.36]	0.1%	10.0%
Huang et al. 2006	101	327	0.31	[0.26; 0.36]	0.1%	10.0%
Hua et al. 1999	1	1543	0.00	[0.00; 0.00]	99.4%	10.5%
Han et al. 2018	2	54	0.04	[0.00; 0.13]	0.1%	10.0%
Fu et al. 2008	3	45	0.07	[0.01; 0.18]	0.0%	9.6%
Chu et al. 2006	6	144	0.04	[0.02; 0.09]	0.2%	10.3%
**Fixed effect model**		**3083**	**0.00**	**[0.00; 0.00]**	**100.0%**	**--**
**Random effects model**			**0.13**	**[0.05; 0.20]**	**--**	**100.0%**

Heterogeneity: $I^2 = 98\%$, $\tau^2 = 0.0148$, $p < 0.01$

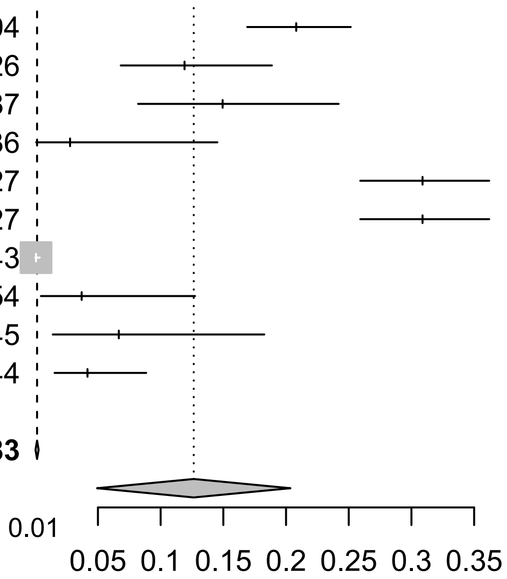

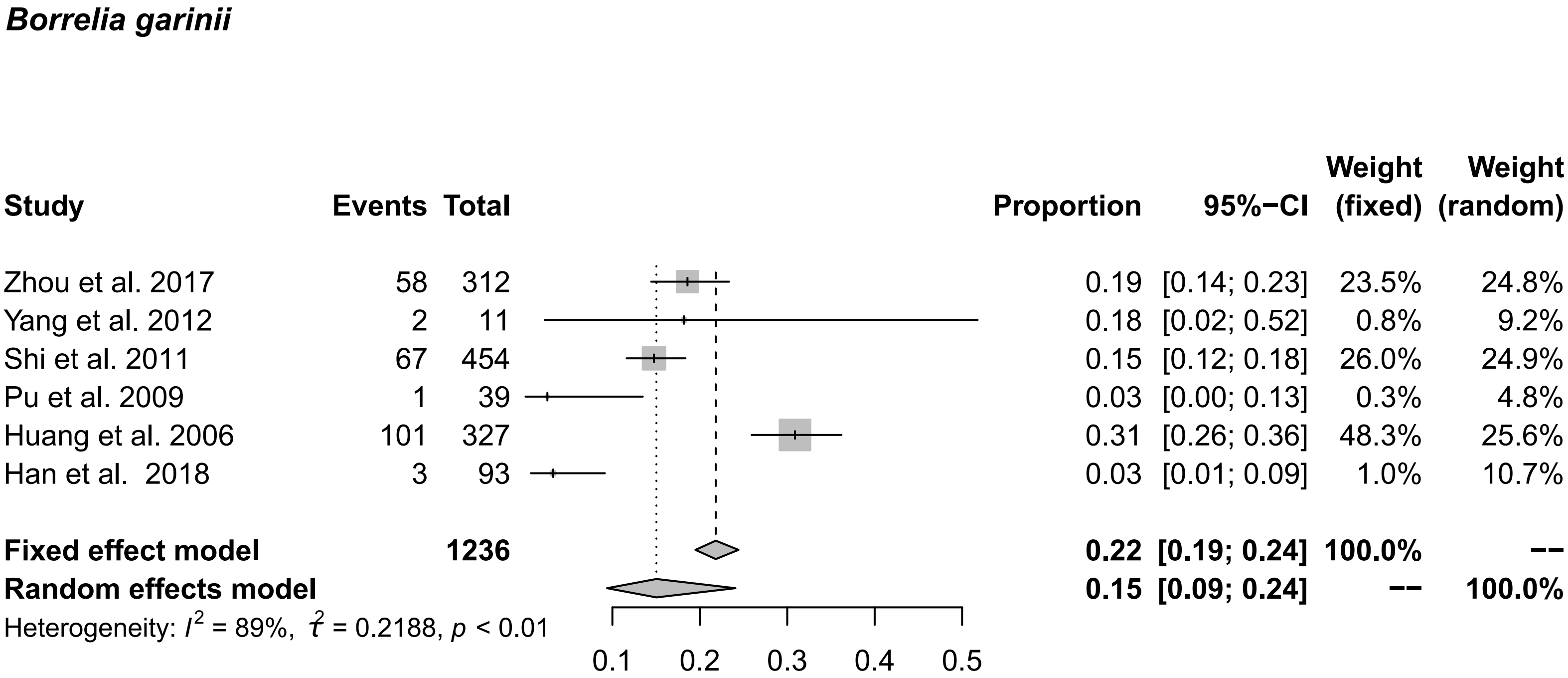
Borrelia garinii
Study
Events
Total
Proportion
95%−CI
Weight (fixed)
Weight (random)
Zhou et al. 2017 58 312 0.19 [0.14; 0.23] 23.5% 24.8%
Yang et al. 2012 2 11 0.18 [0.02; 0.52] 0.8% 9.2%
Shi et al. 2011 67 454 0.15 [0.12; 0.18] 26.0% 24.9%
Pu et al. 2009 1 39 0.03 [0.00; 0.13] 0.3% 4.8%
Huang et al. 2006 101 327 0.31 [0.26; 0.36] 48.3% 25.6%
Han et al. 2018 3 93 0.03 [0.01; 0.09] 1.0% 10.7%
Fixed effect model 1236 0.22 [0.19; 0.24] 100.0% --
Random effects model 0.15 [0.09; 0.24] -- 100.0%
Heterogeneity: I2 = 89%, τ2 = 0.2188, p < 0.01
0.1
0.2
0.3
0.4
0.5

Uncharacterised *Borreliella*

Study	Events	Total	Proportion	95%−CI	Weight (fixed)	Weight (random)
Wan et al. 1998	3	73	0.04	[0.01; 0.12]	91.9%	91.9%
Pukhovskaya et al. 2019	2	17	0.12	[0.01; 0.36]	8.1%	8.1%
**Fixed effect model**		**90**	**0.05**	**[0.00; 0.09]**	**100.0%**	**--**
**Random effects model**			**0.05**	**[0.00; 0.09]**	**--**	**100.0%**

Heterogeneity: $I^2 = 0\%$, $\tau^2 = 0$, $p = 0.35$

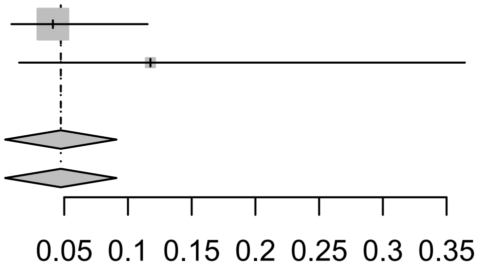

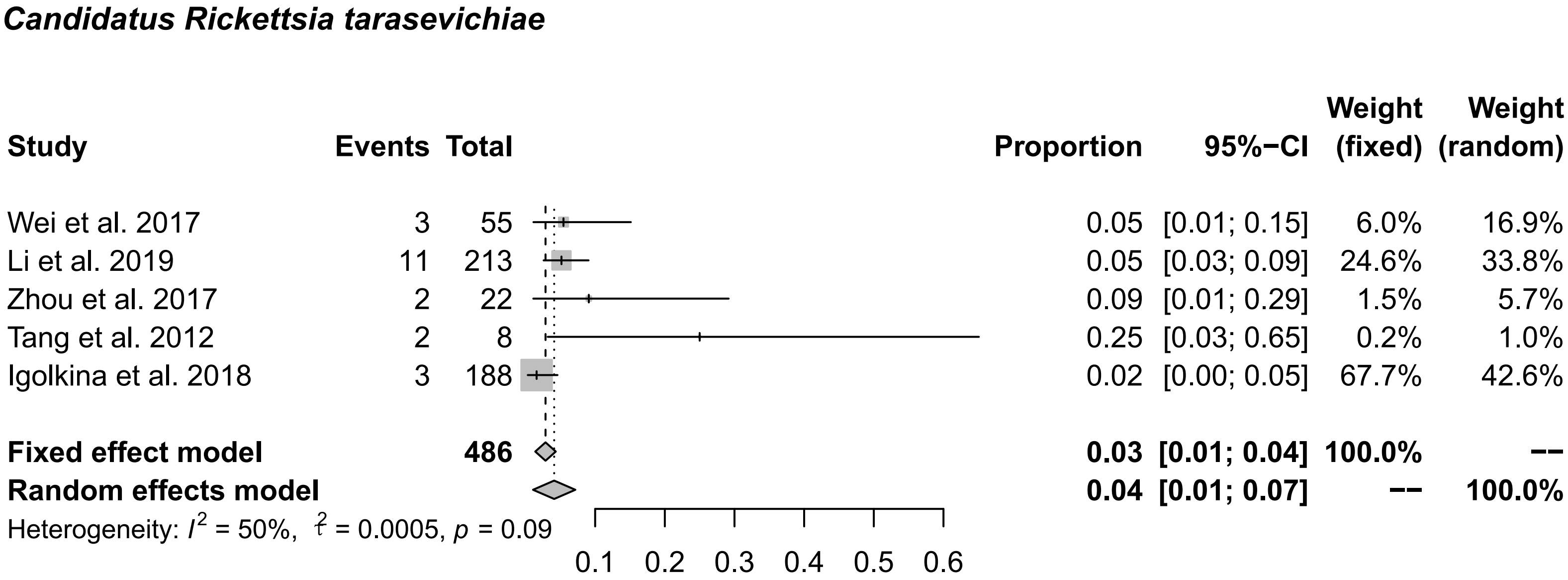
Candidatus Rickettsia tarasevichiae
Study
Events
Total
Proportion
95%−CI
Weight (fixed)
Weight (random)
Wei et al. 2017 3 55 0.05 [0.01; 0.15] 6.0% 16.9%
Li et al. 2019 11 213 0.05 [0.03; 0.09] 24.6% 33.8%
Zhou et al. 2017 2 22 0.09 [0.01; 0.29] 1.5% 5.7%
Tang et al. 2012 2 8 0.25 [0.03; 0.65] 0.2% 1.0%
Igolkina et al. 2018 3 188 0.02 [0.00; 0.05] 67.7% 42.6%
Fixed effect model 486 0.03 [0.01; 0.04] 100.0% --
Random effects model 0.04 [0.01; 0.07] -- 100.0%
Heterogeneity: I^2 = 50%, τ^2 = 0.0005, p = 0.09
0.1 0.2 0.3 0.4 0.5 0.6

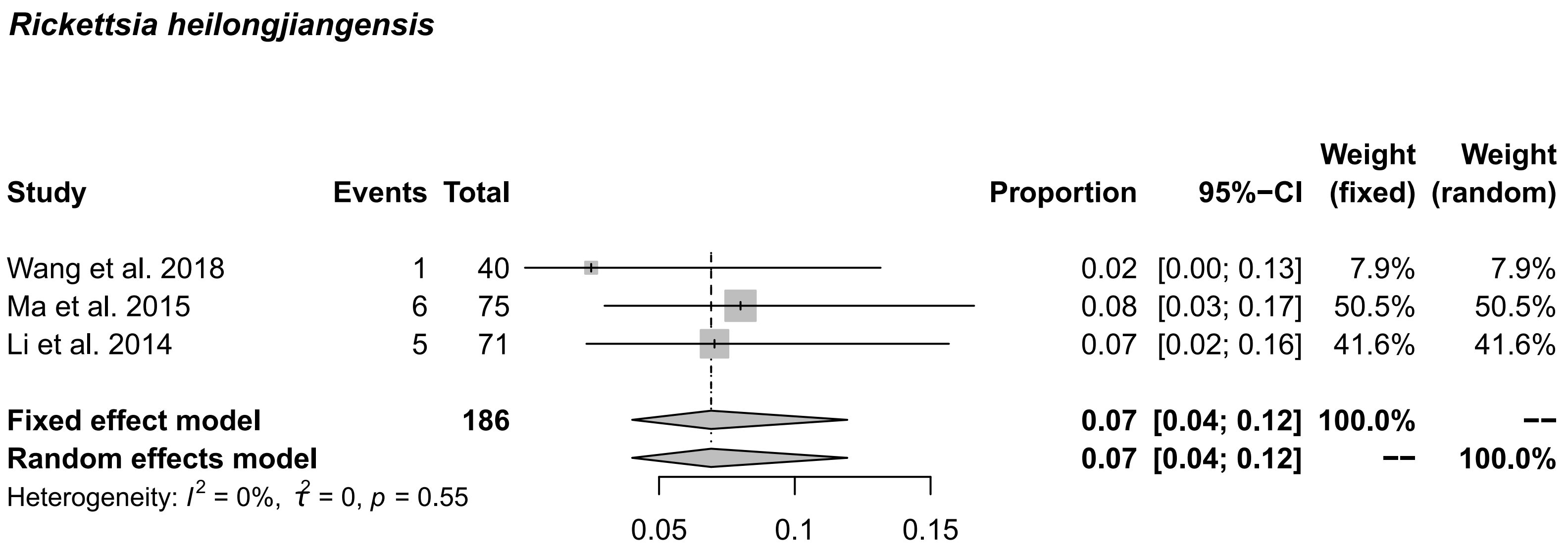
Rickettsia heilongjiangensis
Study Events Total Proportion 95%−CI Weight (fixed) Weight (random)
Wang et al. 2018 1 40 0.02 [0.00; 0.13] 7.9% 7.9%
Ma et al. 2015 6 75 0.08 [0.03; 0.17] 50.5% 50.5%
Li et al. 2014 5 71 0.07 [0.02; 0.16] 41.6% 41.6%
Fixed effect model 186 0.07 [0.04; 0.12] 100.0% --
Random effects model 0.07 [0.04; 0.12] -- 100.0%
Heterogeneity: I^2 = 0%, τ^2 = 0, p = 0.55
0.05
0.1
0.15

Rickettsia raoultii

Study	Events	Total	Proportion	95%−CI	Weight (fixed)	Weight (random)
Ye et al. 2018	866	1054	0.82	[0.80; 0.84]	19.1%	4.3%
Wen et al. 2013	71	626	0.11	[0.09; 0.14]	11.3%	4.3%
Ma et al. 2015	3	75	0.04	[0.01; 0.11]	1.4%	4.2%
Li et al. 2019	41	211	0.19	[0.14; 0.25]	3.8%	4.2%
Han et al. 2018	1	54	0.02	[0.00; 0.10]	1.0%	4.1%
Fu et al. 2015	17	20	0.85	[0.62; 0.97]	0.4%	3.9%
Feng et al. 2014	14	60	0.23	[0.13; 0.36]	1.1%	4.1%
Cheng et al. 2019	329	623	0.53	[0.49; 0.57]	11.3%	4.3%
Cheng et al. 2017	321	625	0.51	[0.47; 0.55]	11.3%	4.3%
Cheng et al. 2015	62	94	0.66	[0.55; 0.75]	1.7%	4.2%
Chen et al. 2015	9	200	0.04	[0.02; 0.08]	3.6%	4.2%
Cao et al. 2014	14	62	0.23	[0.13; 0.35]	1.1%	4.1%
Zhou et al. 2017	11	22	0.50	[0.28; 0.72]	0.4%	3.9%
Yang et al. 2016	26	66	0.39	[0.28; 0.52]	1.2%	4.2%
Igolkina et al. 2018	136	188	0.72	[0.65; 0.79]	3.4%	4.2%
Liu et al. 2016	9	204	0.04	[0.02; 0.08]	3.7%	4.2%
zhao et al. 2019	3	42	0.07	[0.01; 0.19]	0.8%	4.1%
Wen et al. 2014	30	93	0.32	[0.23; 0.43]	1.7%	4.2%
Shao et al. 2020	77	165	0.47	[0.39; 0.55]	3.0%	4.2%
Liu et al. 2020	3	46	0.07	[0.01; 0.18]	0.8%	4.1%
Jia et al. 2014	3	75	0.04	[0.01; 0.11]	1.4%	4.2%
Tian et al. 2012	9	200	0.04	[0.02; 0.08]	3.6%	4.2%
Wang et al. 2018	2	40	0.05	[0.01; 0.17]	0.7%	4.1%
Cao et al. 2008	25	676	0.04	[0.02; 0.05]	12.2%	4.3%
**Fixed effect model**		**5521**	**0.34**	**[0.33; 0.35]**	**100.0%**	**--**
**Random effects model**			**0.25**	**[0.13; 0.39]**	**--**	**100.0%**

Heterogeneity: $I^2 = 99\%$, $\tau^2 = 0.1409$, $p = 0$

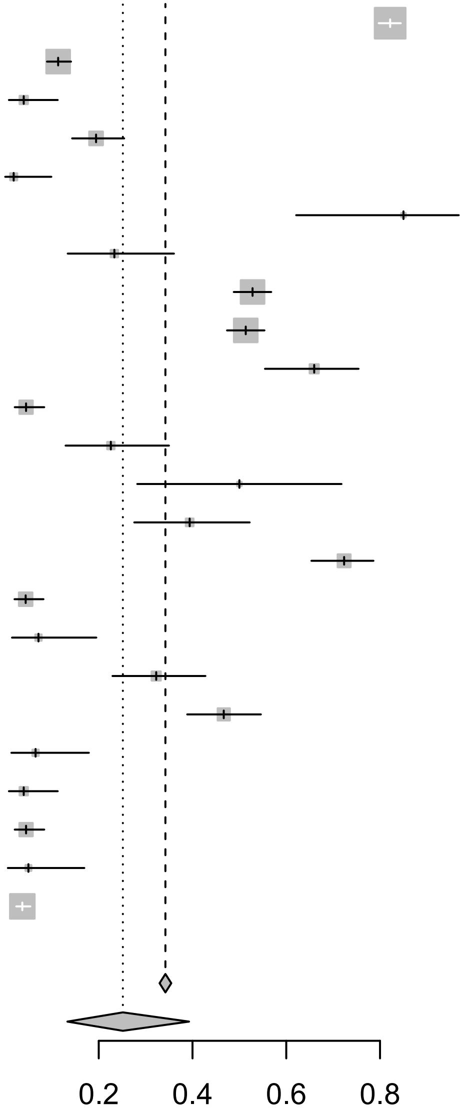

Rickettsia sibirica

Study	Events	Total	Proportion	95%−CI	Weight (fixed)	Weight (random)
Yang et al. 2012	4	34	0.12	[0.03; 0.27]	1.7%	12.5%
Liu et al. 1987	42	312	0.13	[0.10; 0.18]	14.2%	24.6%
Gao et al. 2019	5	158	0.03	[0.01; 0.07]	27.4%	26.4%
Cao et al. 2008	47	676	0.07	[0.05; 0.09]	55.6%	27.4%
zhao et al. 2019	13	42	0.31	[0.18; 0.47]	1.0%	9.1%
**Fixed effect model**		**1222**	**0.07**	**[0.06; 0.09]**	**100.0%**	**--**
**Random effects model**			**0.10**	**[0.05; 0.15]**	**--**	**100.0%**

Heterogeneity: $I^2 = 87\%$, $\tau^2 = 0.0024$, $p < 0.01$

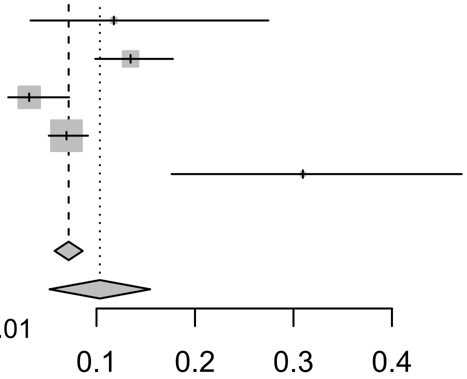

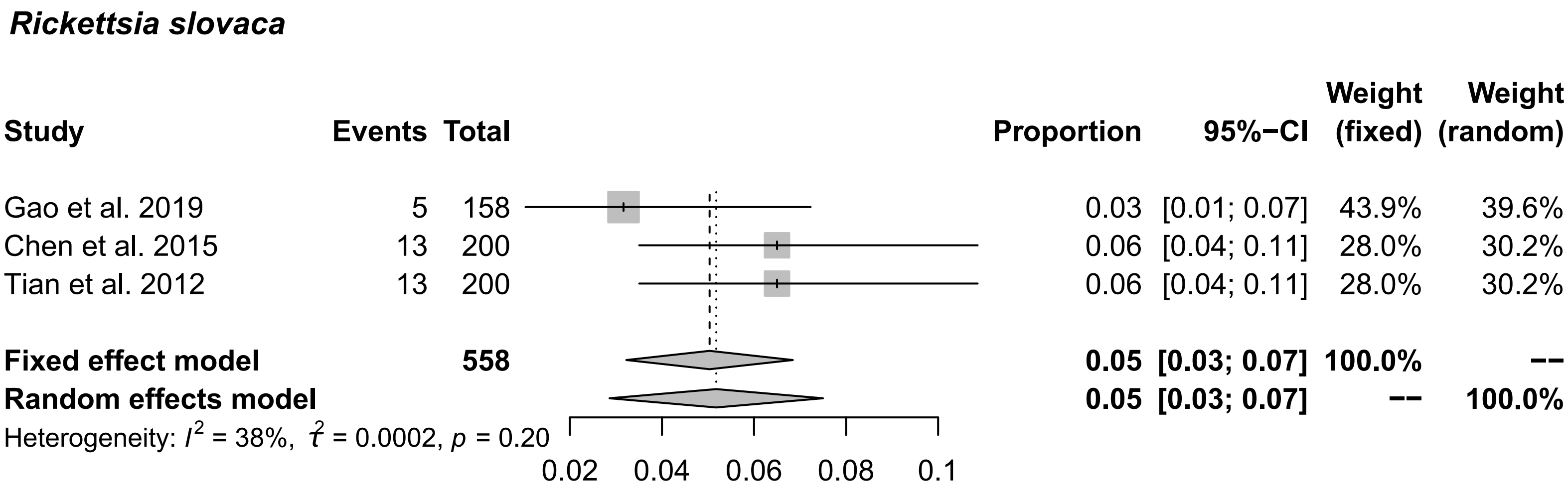
Rickettsia slovaca
Study
Events
Total
Proportion
95%−CI
Weight (fixed)
Weight (random)
Gao et al. 2019 5 158 0.03 [0.01; 0.07] 43.9% 39.6%
Chen et al. 2015 13 200 0.06 [0.04; 0.11] 28.0% 30.2%
Tian et al. 2012 13 200 0.06 [0.04; 0.11] 28.0% 30.2%
Fixed effect model 558 0.05 [0.03; 0.07] 100.0% --
Random effects model 0.05 [0.03; 0.07] -- 100.0%
Heterogeneity: I2 = 38%, τ2 = 0.0002, p = 0.20
0.02
0.04
0.06
0.08
0.1

Uncharacterised *Rickettsia*

Study	Events	Total	Proportion	95%−CI	Weight (fixed)	Weight (random)
Wu et al. 2012	6	127	0.05	[0.02; 0.10]	1.1%	13.0%
Wang et al. 2016	1	92	0.01	[0.00; 0.06]	0.2%	3.3%
Huang et al. 2006	95	327	0.29	[0.24; 0.34]	22.4%	27.6%
Huang et al. 2006	95	327	0.29	[0.24; 0.34]	22.4%	27.6%
Han et al. 2014	195	495	0.39	[0.35; 0.44]	53.9%	28.5%
**Fixed effect model**		**1368**	**0.33**	**[0.31; 0.36]**	**100.0%**	**--**
**Random effects model**			**0.22**	**[0.15; 0.33]**	**--**	**100.0%**

Heterogeneity: $I^2 = 92\%$, $\tau^2 = 0.1264$, $p < 0.01$

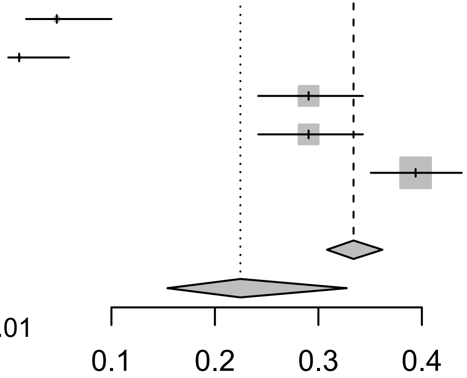

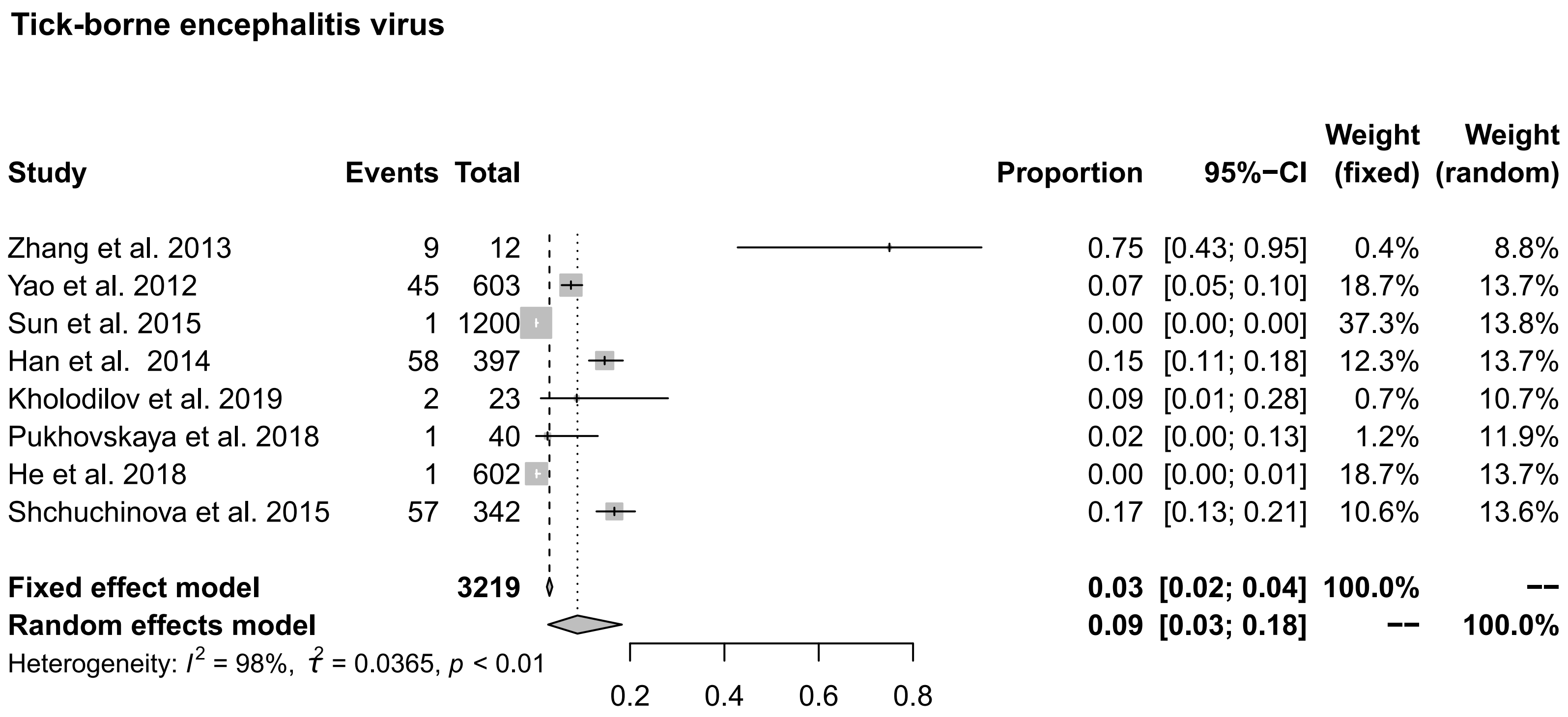
Tick-borne encephalitis virus
Study
Events
Total
Proportion
95%−CI
Weight (fixed)
Weight (random)
Zhang et al. 2013 9 12 0.75 [0.43; 0.95] 0.4% 8.8%
Yao et al. 2012 45 603 0.07 [0.05; 0.10] 18.7% 13.7%
Sun et al. 2015 1 1200 0.00 [0.00; 0.00] 37.3% 13.8%
Han et al. 2014 58 397 0.15 [0.11; 0.18] 12.3% 13.7%
Kholodilov et al. 2019 2 23 0.09 [0.01; 0.28] 0.7% 10.7%
Pukhovskaya et al. 2018 1 40 0.02 [0.00; 0.13] 1.2% 11.9%
He et al. 2018 1 602 0.00 [0.00; 0.01] 18.7% 13.7%
Shchuchinova et al. 2015 57 342 0.17 [0.13; 0.21] 10.6% 13.6%
Fixed effect model 3219 0.03 [0.02; 0.04] 100.0% --
Random effects model 0.09 [0.03; 0.18] -- 100.0%
Heterogeneity: I^2 = 98%, τ^2 = 0.0365, p < 0.01
0.2
0.4
0.6
0.8

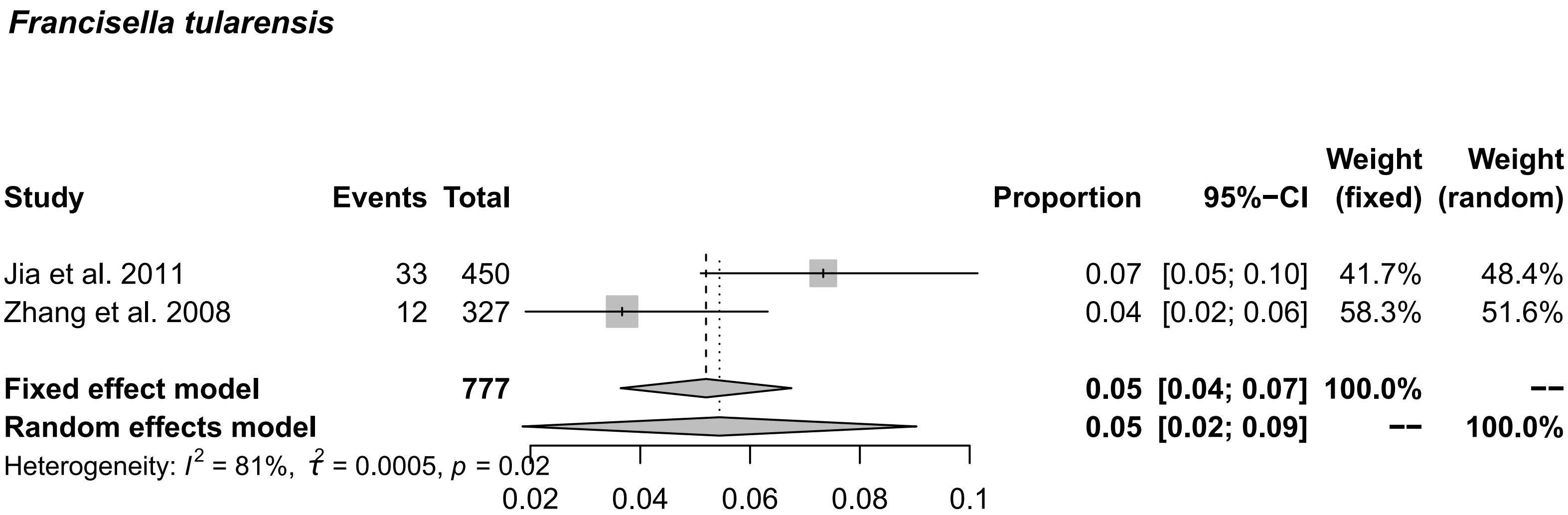
Francisella tularensis
Study
Events
Total
Proportion
95%−CI
Weight (fixed)
Weight (random)
Jia et al. 2011 33 450 0.07 [0.05; 0.10] 41.7% 48.4%
Zhang et al. 2008 12 327 0.04 [0.02; 0.06] 58.3% 51.6%
Fixed effect model 777 0.05 [0.04; 0.07] 100.0% --
Random effects model 0.05 [0.02; 0.09] -- 100.0%
Heterogeneity: I^2 = 81%, τ^2 = 0.0005, p = 0.02
0.02
0.04
0.06
0.08
0.1

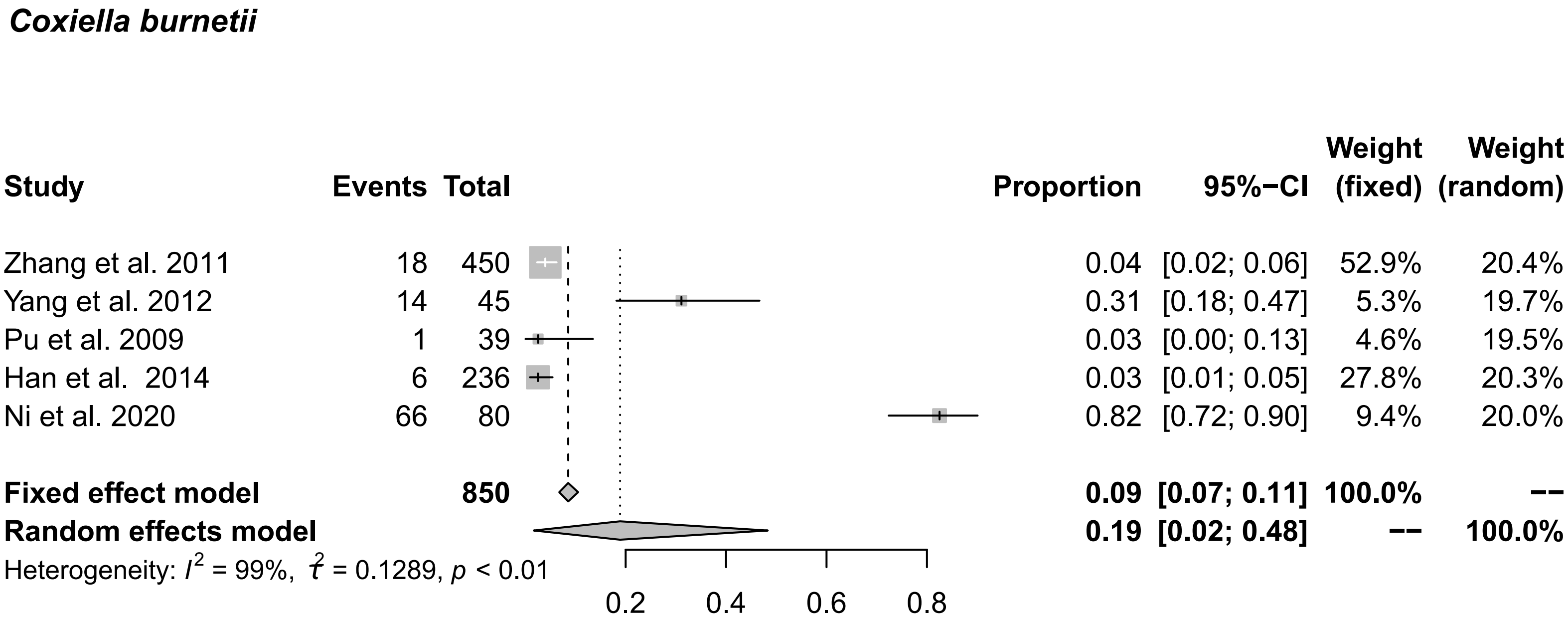
Coxiella burnetii
Study
Events
Total
Proportion
95%−CI
Weight (fixed)
Weight (random)
Zhang et al. 2011 18 450 0.04 [0.02; 0.06] 52.9% 20.4%
Yang et al. 2012 14 45 0.31 [0.18; 0.47] 5.3% 19.7%
Pu et al. 2009 1 39 0.03 [0.00; 0.13] 4.6% 19.5%
Han et al. 2014 6 236 0.03 [0.01; 0.05] 27.8% 20.3%
Ni et al. 2020 66 80 0.82 [0.72; 0.90] 9.4% 20.0%
Fixed effect model 850 0.09 [0.07; 0.11] 100.0% --
Random effects model 0.19 [0.02; 0.48] -- 100.0%
Heterogeneity: I^2 = 99%, τ^2 = 0.1289, p < 0.01
0.2
0.4
0.6
0.8

***Bartonella* sp. Koshimizu 6-1**

Study	Events	Total	Proportion	95%−CI	Weight (fixed)	Weight (random)
Cheng et al. 2017	11	188	0.06	[0.03; 0.10]	78.4%	78.4%
Yang et al. 2016	5	66	0.08	[0.03; 0.17]	21.6%	21.6%
**Fixed effect model**		**254**	**0.06**	**[0.03; 0.09]**	**100.0%**	**−−**
**Random effects model**			**0.06**	**[0.03; 0.09]**	**−−**	**100.0%**

Heterogeneity: $I^2 = 0\%$, $\tau^2 = 0$, $p = 0.64$

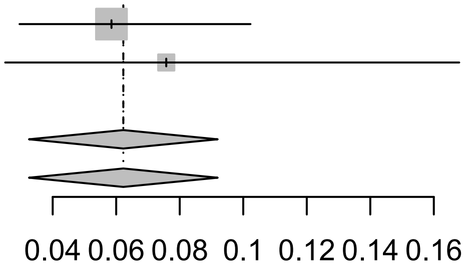

Uncharacterised *Bartonella*

Study	Events	Total	Proportion	95%−CI	Weight (fixed)	Weight (random)
Ye et al. 2018	45	1054	0.04	[0.03; 0.06]	65.4%	65.4%
Ju et al. 2018	22	583	0.04	[0.02; 0.06]	31.8%	31.8%
Han et al. 2014	2	107	0.02	[0.00; 0.07]	2.8%	2.8%
**Fixed effect model**		**1744**	**0.04**	**[0.03; 0.05]**	**100.0%**	**--**
**Random effects model**			**0.04**	**[0.03; 0.05]**	**--**	**100.0%**

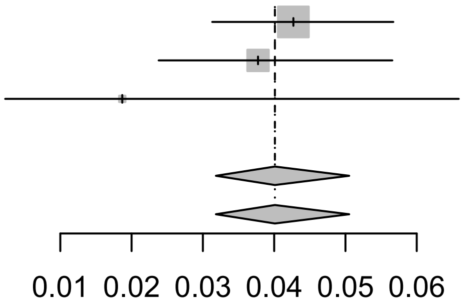

Heterogeneity: $I^2 = 0\%$, $\tau^2 = 0$, $p = 0.48$
