# Supplementary material for: Distribution of Dermacentor silvarum and Associated Pathogens: Meta-Analysis of Global Published Data and a Field Survey in China
Source: Int J Environ Res Public Health. 2021 Apr 22;18(9):4430. doi: 10.3390/ijerph18094430 (PMC8122522; doi:10.3390/ijerph18094430)
Supplement: Supplementary file 1 [file ijerph-18-04430-s001.zip › Supplementary -pdf/FigureS6.pdf]

Figure S6. Geographic distribution of each *Dermacentor silvarum*-associated agent in China. (A) Anaplasmataceae family. (B) spotted fever group rickettsiae. (C) *Babesia*. (D) *Bartonella*. (E) *Borrelia*. (F) viruses. (G) other agents. Dark gray represents the county level administrative regions, light gray represents the prefecture level administrative regions.

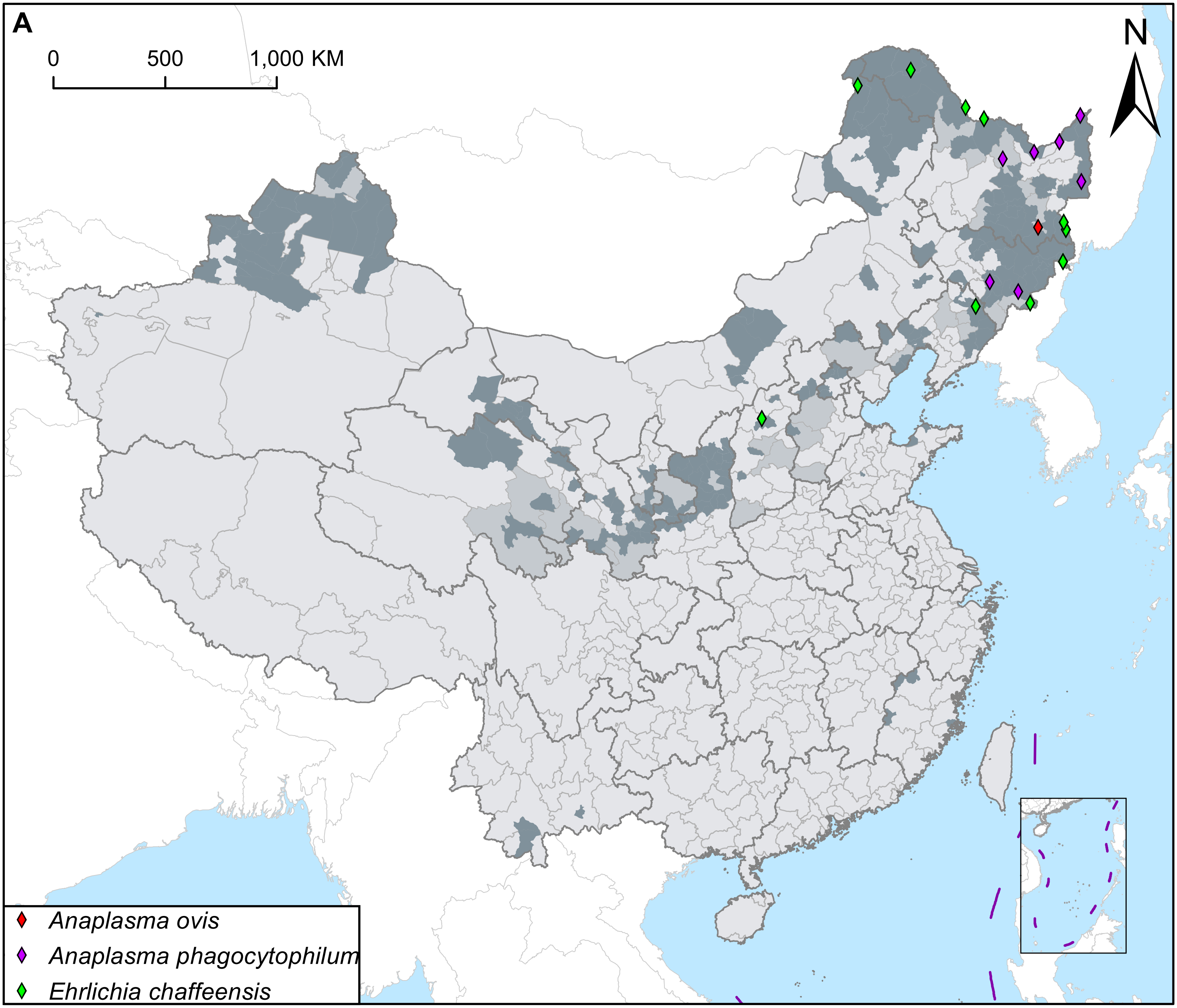

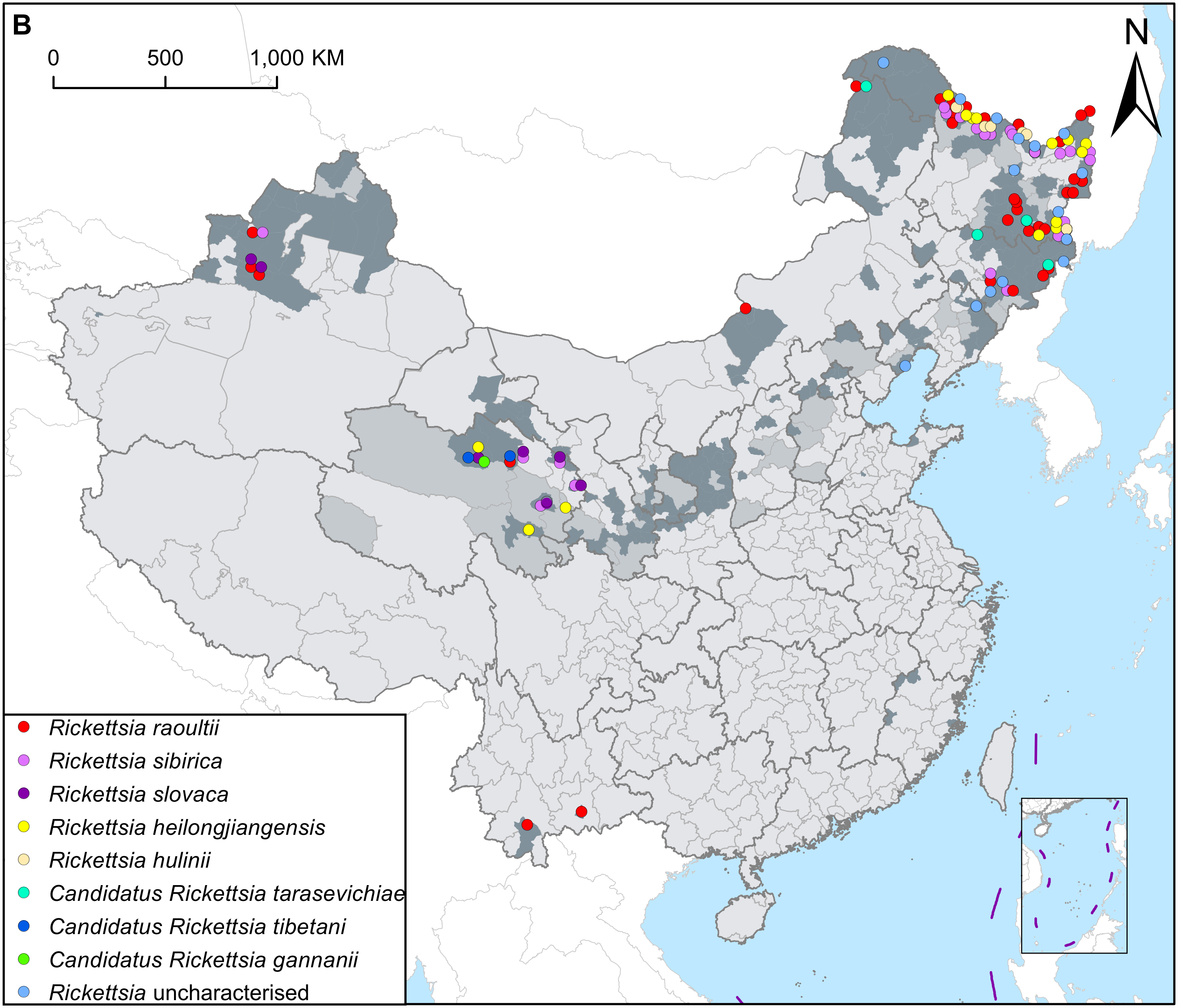
B
0
500
1,000 KM
N
Rickettsia raoultii
Rickettsia sibirica
Rickettsia slovaca
Rickettsia heilongjiangensis
Rickettsia hulinii
Candidatus Rickettsia tarasevichiae
Candidatus Rickettsia tibetani
Candidatus Rickettsia gannanii
Rickettsia uncharacterised

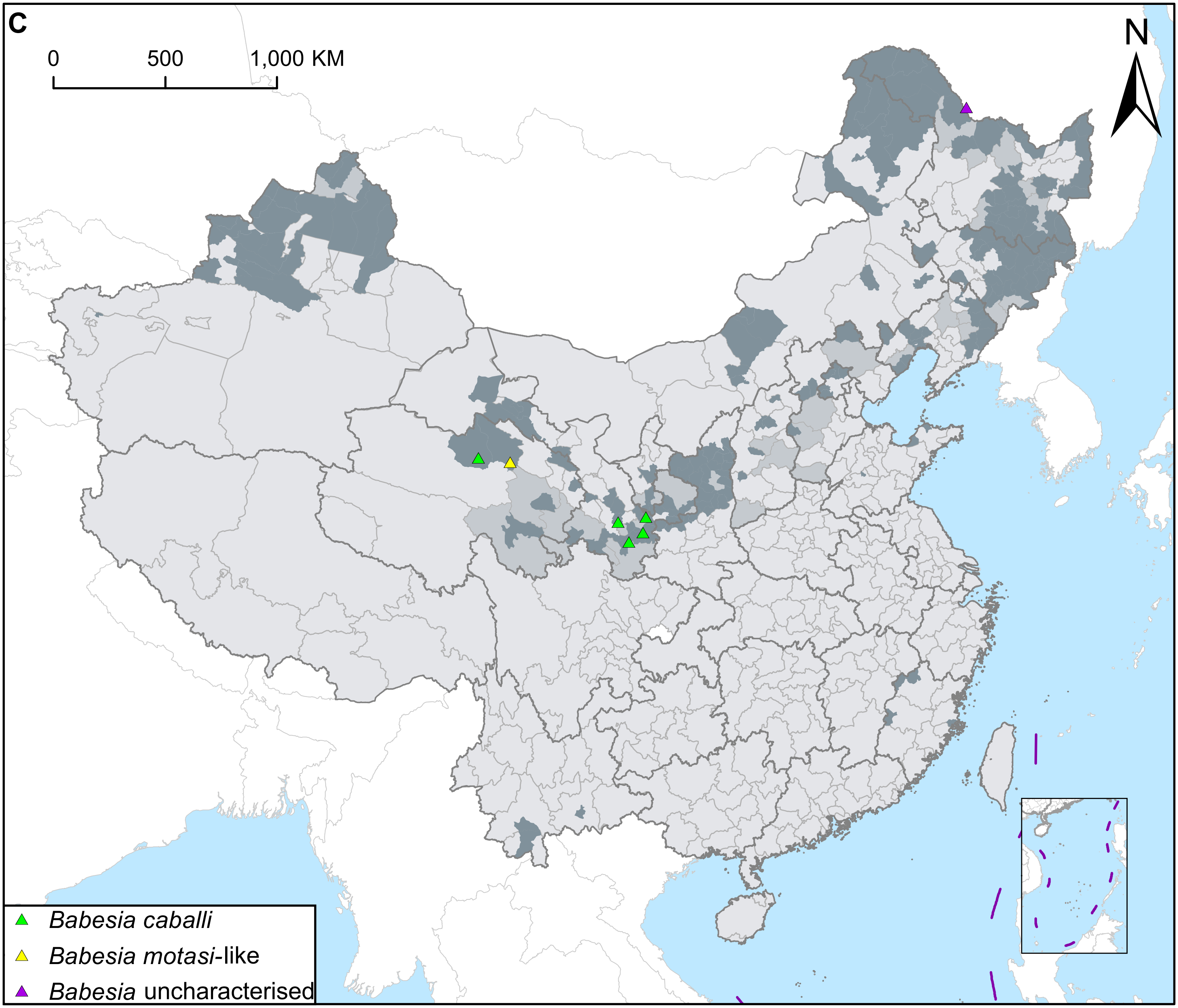
C
0
500
1,000 KM
N
Babesia caballi
Babesia motasi-like
Babesia uncharacterised

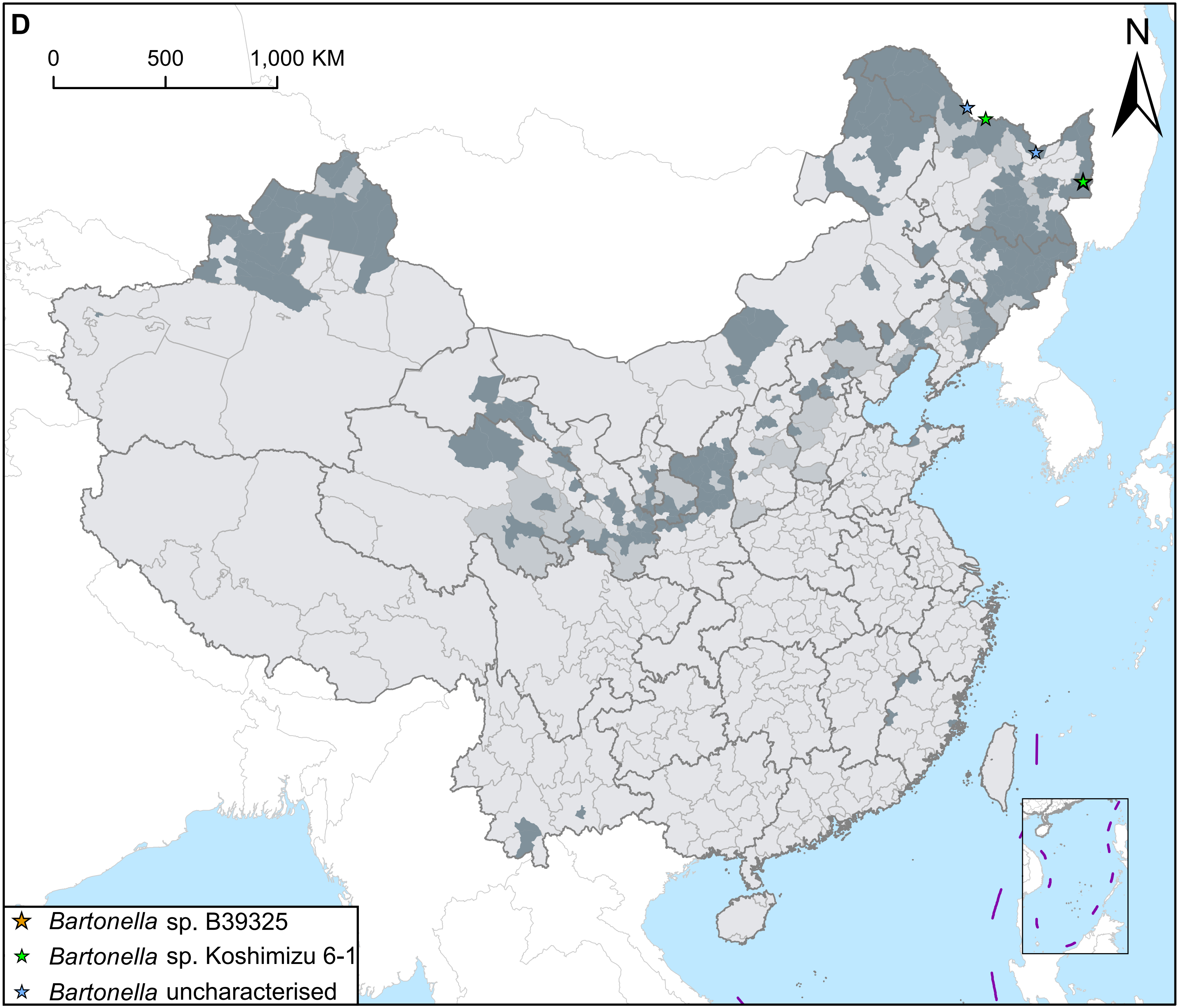

D
0
500
1,000 KM
N
Bartonella sp. B39325
Bartonella sp. Koshimizu 6-1
Bartonella uncharacterised

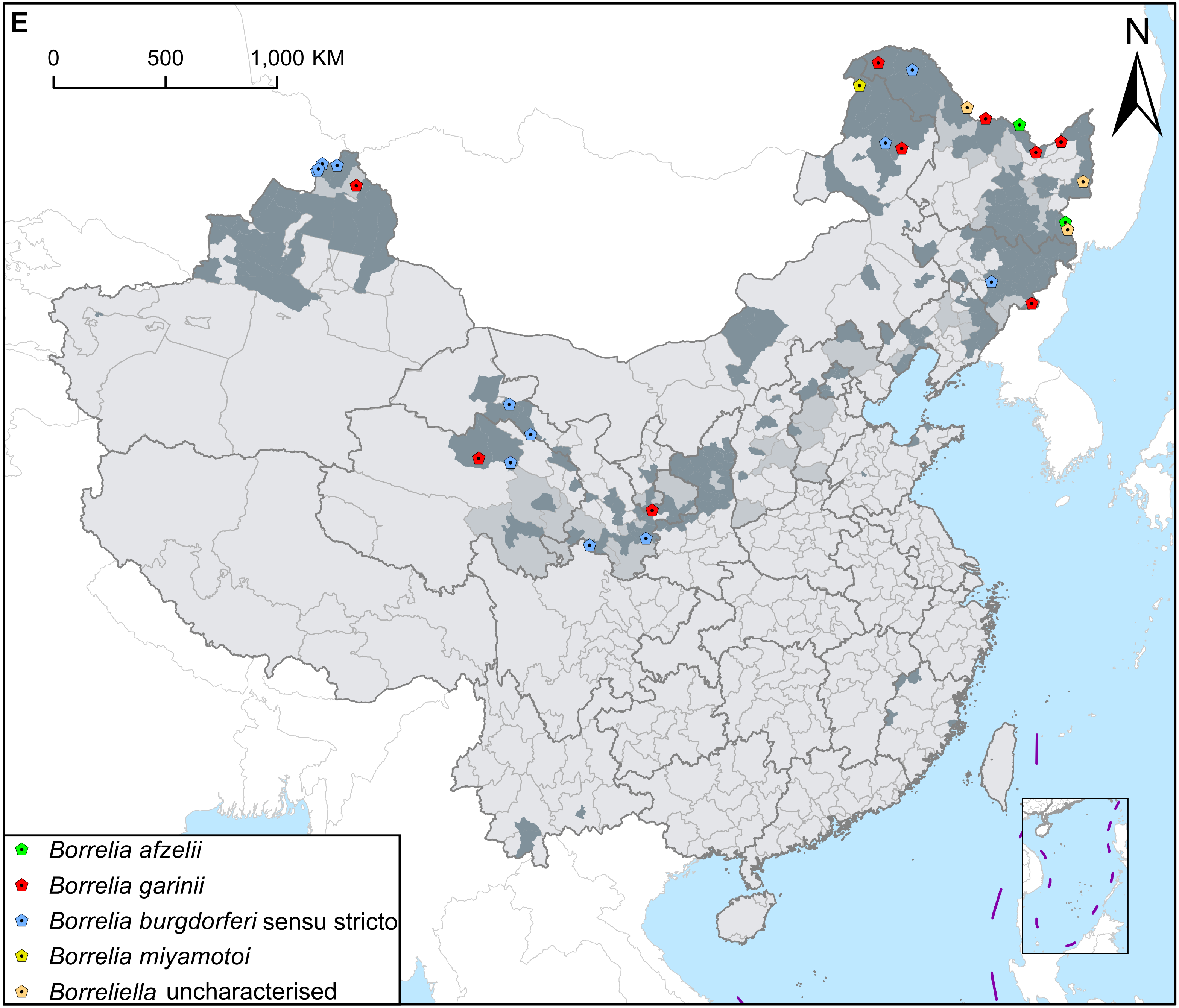
E
0
500
1,000 KM
N
Borrelia afzelii
Borrelia garinii
Borrelia burgdorferi sensu stricto
Borrelia miyamotoi
Borreliella uncharacterised

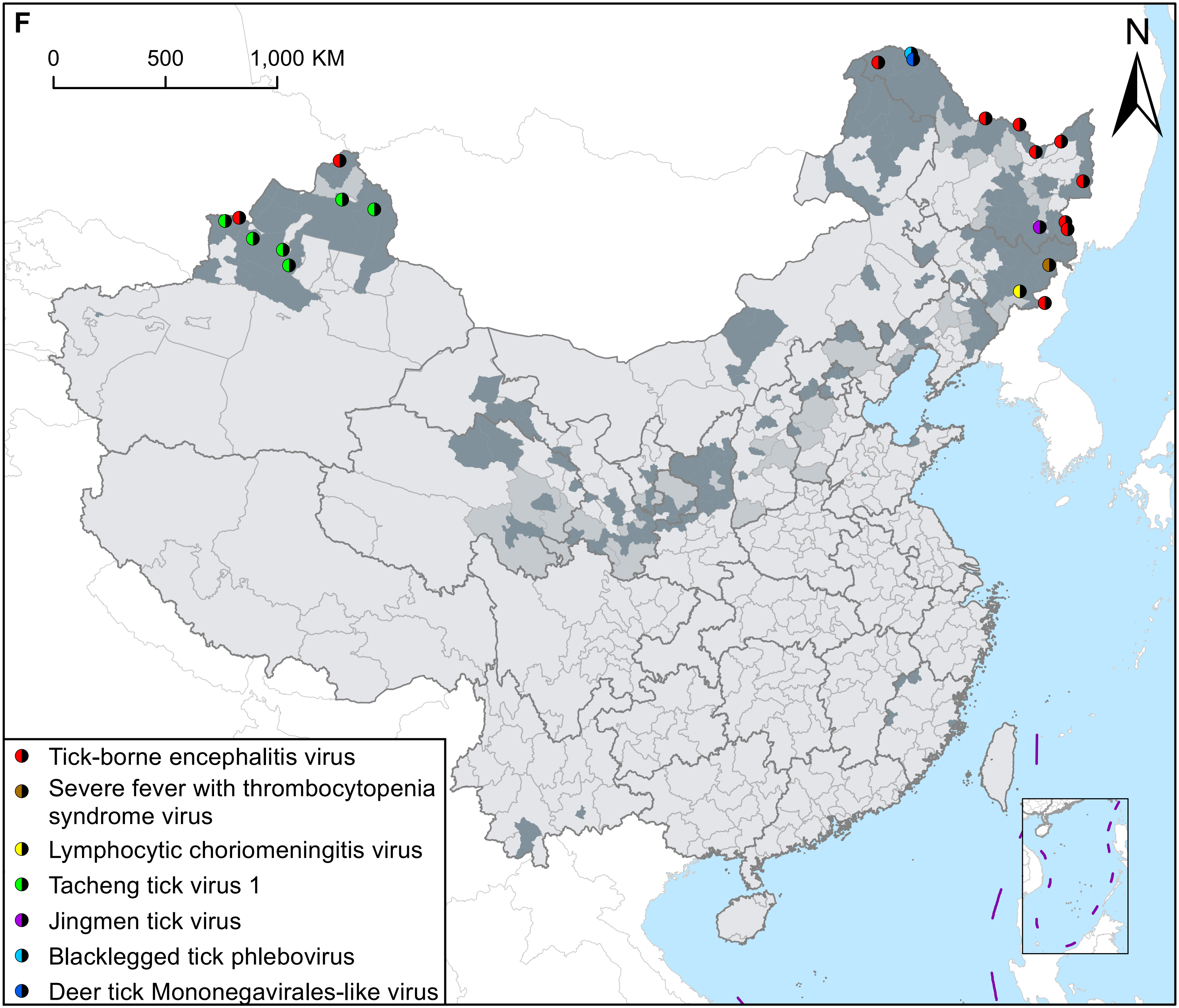
F
0
500
1,000 KM
N
Tick-borne encephalitis virus
Severe fever with thrombocytopenia syndrome virus
Lymphocytic choriomeningitis virus
Tacheng tick virus 1
Jingmen tick virus
Blacklegged tick phlebovirus
Deer tick Mononegavirales-like virus

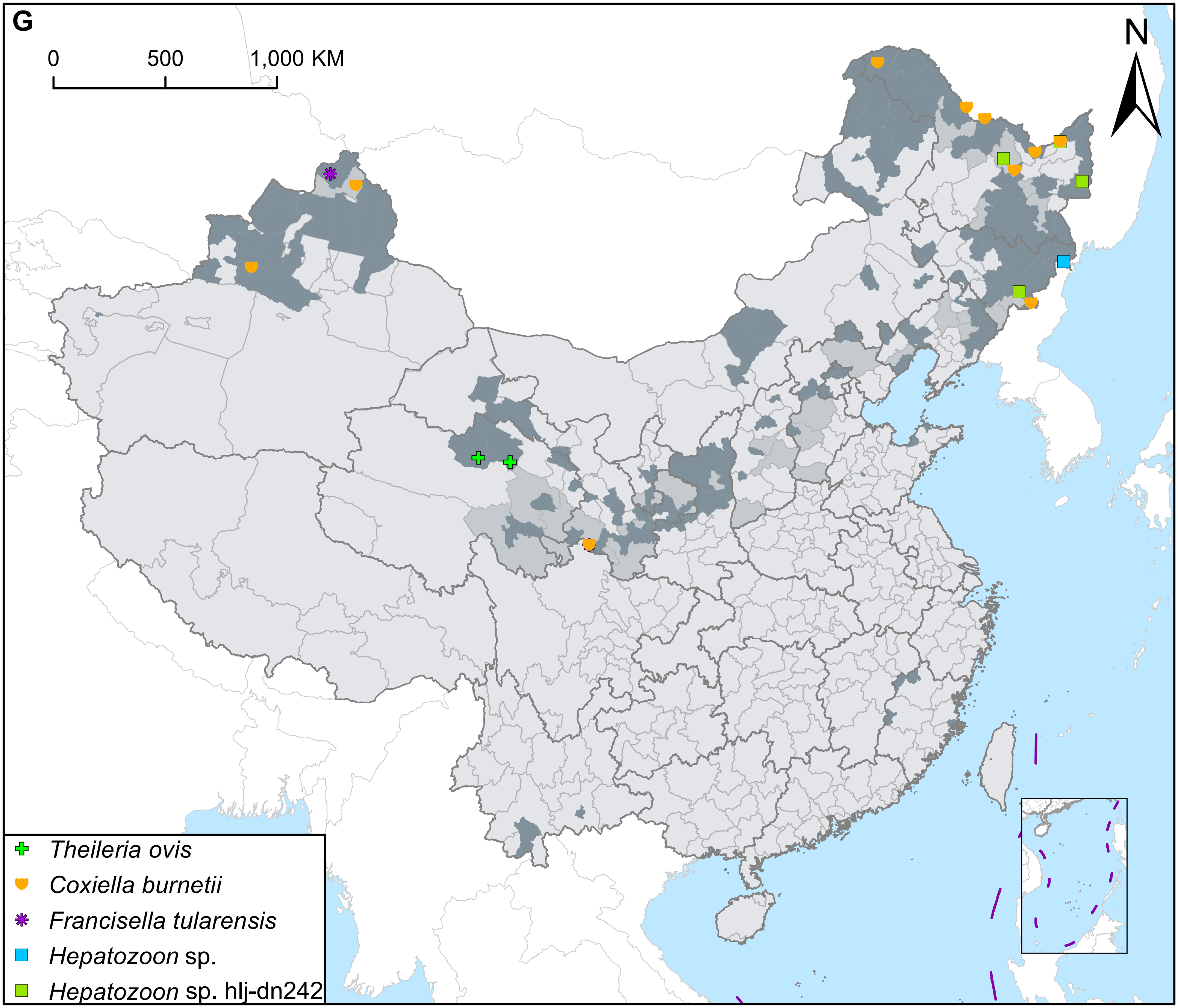
G
0
500
1,000 KM
N
Theileria ovis
Coxiella burnetii
Francisella tularensis
Hepatozoon sp.
Hepatozoon sp. hlj-dn242
