# Supplementary material for: Distribution of Dermacentor silvarum and Associated Pathogens: Meta-Analysis of Global Published Data and a Field Survey in China
Source: Int J Environ Res Public Health. 2021 Apr 22;18(9):4430. doi: 10.3390/ijerph18094430 (PMC8122522; doi:10.3390/ijerph18094430)
Supplement: Supplementary file 1 [file ijerph-18-04430-s001.zip › Supplementary -pdf/TableS3.pdf]

Table S2. Prevalence and pathogenicity of *Dermacentor silvarum*-associated agents.

If there was only one study included for a certain species of *D. silvarum*-associated agent, the positive rate was calculated by positive ticks divided by total ticks and without a 95% CI. If the number of studies was two or more, the combined positive rate and 95% CI were estimated by meta-analysis.

	Positive rate (95% CI)	Number of studies
**Anaplasmataceae**		
*Anaplasma ovis**	0.015	1
*Anaplasma phagocytophilum**	0.0614(0.0153-0.1354)	9
Anaplasmataceae bacterium IS136	0.07	1
*Ehrlichia chaffeensis**	0.0903(0.0604-0.1201)	12
Babesia		
Babesia caballi	0.1085(0.0178-0.1992)	2
Babesia motasi-like LT/TZ	0.13	1
*Babesia venatorum**	0.016	1
Babesia uncharacterised	0.0171	1
Bartonella		
Bartonella sp. B39325	0.16	1
Bartonella sp. Koshimizu 6-1	0.0622(0.0325-0.0919)	2
Bartonella uncharacterised	0.0401(0.0318-0.0505)	3
***Borrelia burgdorferi* sensu lato**		
*Borreliella afzelii**	0.0565(0.0417-0.0764)	4
*Borreliella garinii**	0.1503(0.0939-0.2408)	6
Borrelia burgdorferi sensu stricto*	0.1264(0.0493-0.2035)	10
Borreliella uncharacterized	0.0473(0.0037-0.091)	2
Borrelia		
*Borrelia miyamotoi**	0.027	1
Coxiella		
*Coxiella burnetii**	0.1889(0.017-0.4829)	5
Francisella		
*Francisella tularensis**	0.0544(0.0185-0.0903)	2
Theileria		
Theileria ovis	0.108	1
Hepatozoon		
Hepatozoon sp.	0.024	1
Hepatozoon sp. hlj-dn242	0.029	1
**Spotted fever group rickettsiae**		
*Rickettsia heilongjiangensis**	0.0692(0.0401-0.1193)	3

Rickettsia hulinii	0.25	1
*Rickettsia raoultii**	0.2515(0.1331-0.3927)	24
*Rickettsia sibirica**	0.1034(0.0523-0.1546)	5
*Rickettsia slovaca**	0.0528(0.0286-0.075)	3
Rickettsia sp. JL-02	0.05	1
Rickettsia uncharacterised	0.2246(0.1541-0.3274)	5
Candidatus Rickettsia gannanii Y27	0.026	1
*Candidatus Rickettsia tarasevichiae**	0.0412(0.0108-0.0715)	5
Candidatus Rickettsia tibetani	0.022	1
**Viruses**		
Tacheng tick virus 1*	0.118	1
Tick-borne encephalitis virus*	0.0885(0.0262-0.1826)	8
Blacklegged tick phlebovirus	1	1
Deer tick Mononegavirales-like virus	1	1
Jingmen tick virus*	NA	1
Lymphocytic choriomeningitis virus*	0.037	1
Severe fever with thrombocytopenia syndrome virus*	0.005	1

*Pathogenic to humans; CI: Confidence Interval.

NA: The original article did not mention the specific number of tests and positive numbers.
