# Supplementary figures and images for: Distribution of Dermacentor silvarum and Associated Pathogens: Meta-Analysis of Global Published Data and a Field Survey in China
Source: Int J Environ Res Public Health. 2021 Apr 22;18(9):4430. doi: 10.3390/ijerph18094430 (PMC8122522; doi:10.3390/ijerph18094430)

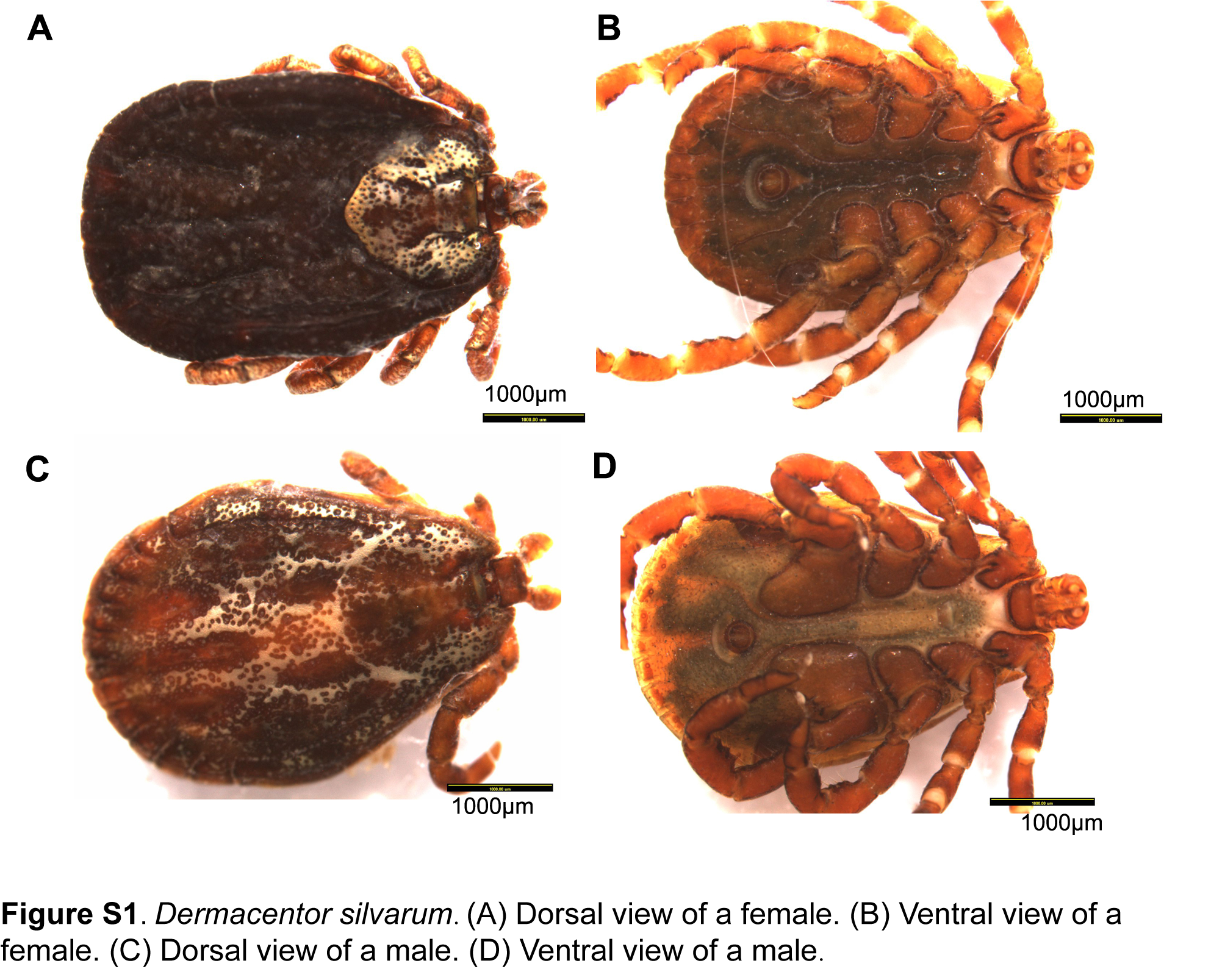

Supplement: Supplementary file 1 [file ijerph-18-04430-s001.zip › Supplementary -pdf/FigureS1.tif]

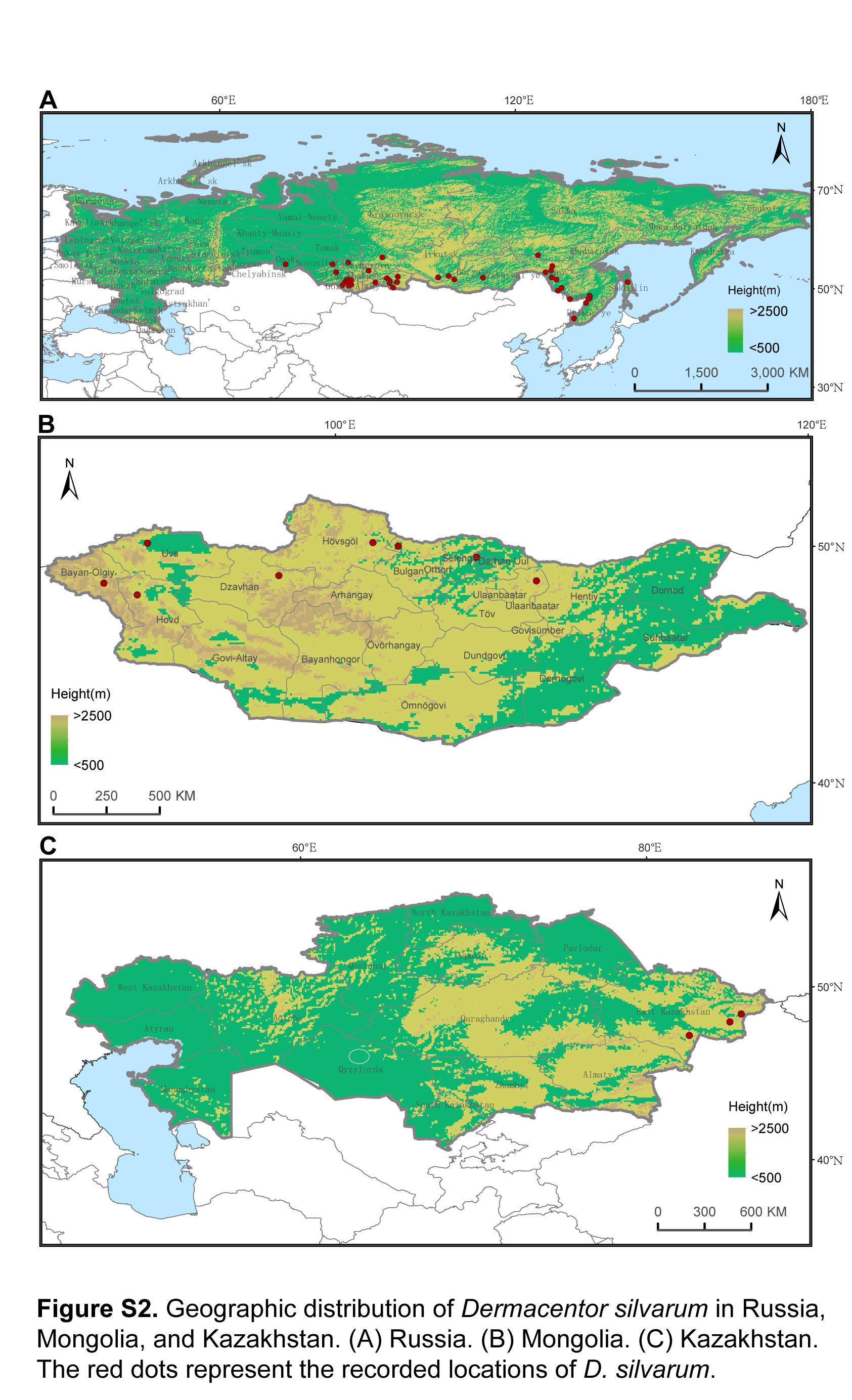

Supplement: Supplementary file 1 [file ijerph-18-04430-s001.zip › Supplementary -pdf/FigureS2.tif]

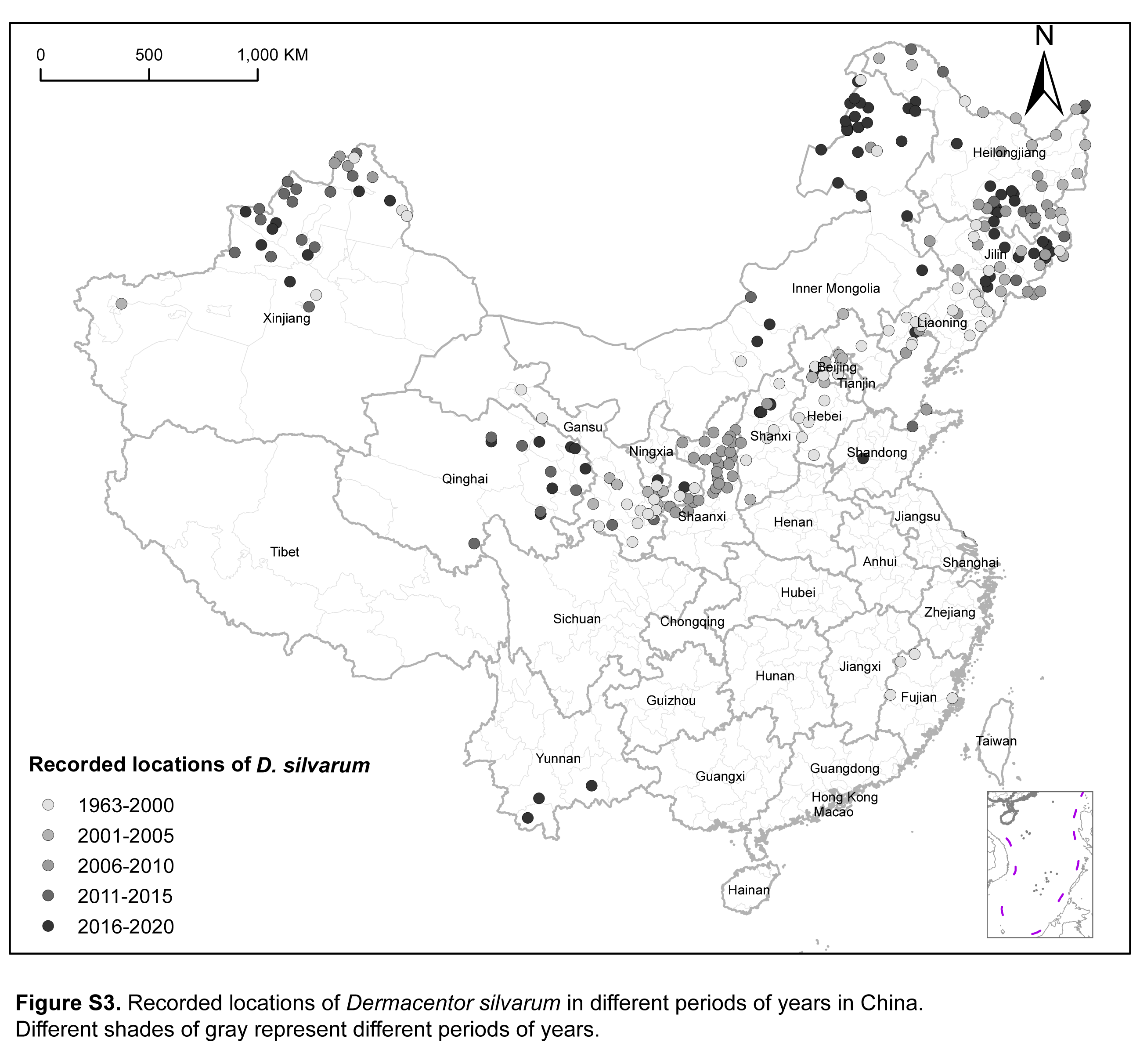

Supplement: Supplementary file 1 [file ijerph-18-04430-s001.zip › Supplementary -pdf/FigureS3.tif]

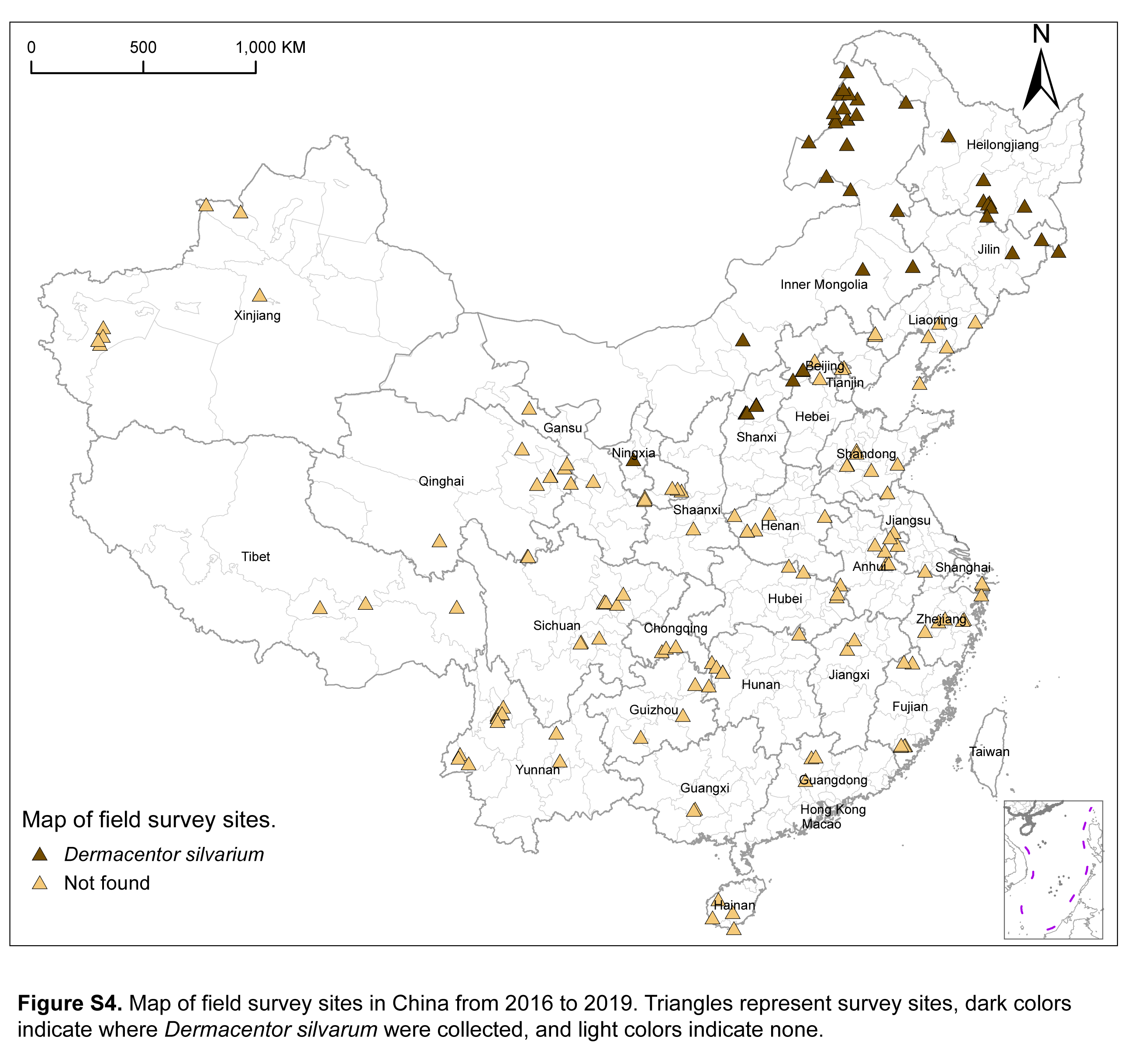

Supplement: Supplementary file 1 [file ijerph-18-04430-s001.zip › Supplementary -pdf/FigureS4.tif]

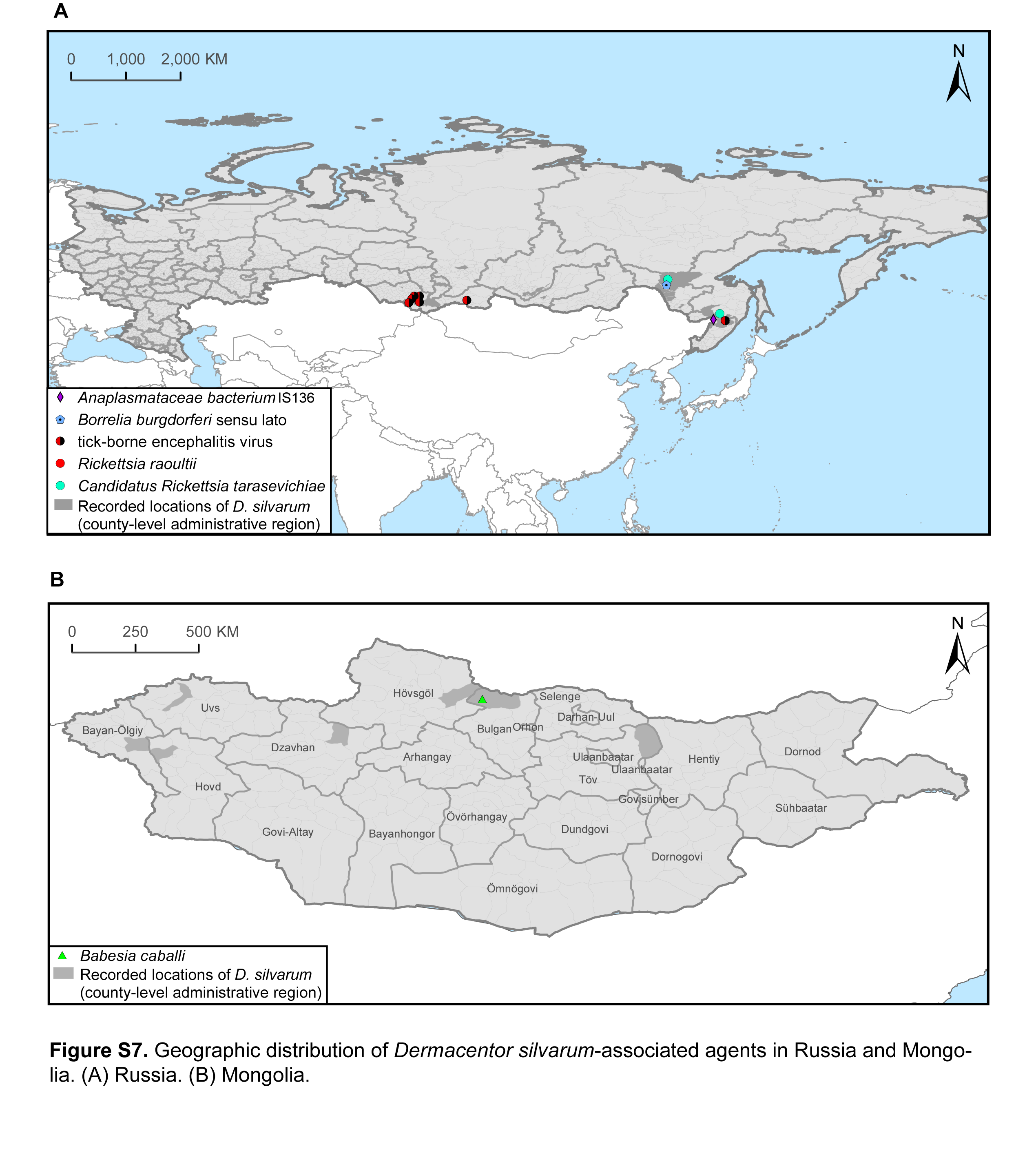

Supplement: Supplementary file 1 [file ijerph-18-04430-s001.zip › Supplementary -pdf/FigureS7.tif]
